# A Robust Marine Collagen Peptide–Agarose 3D Culture System for In Vitro Modeling of Hepatocellular Carcinoma and Anti-Cancer Therapeutic Development

**DOI:** 10.3390/md23100386

**Published:** 2025-09-27

**Authors:** Lata Rajbongshi, Ji-Eun Kim, Jin-Eui Lee, Su-Rin Lee, Seon-Yeong Hwang, Yuna Kim, Young Mi Hong, Sae-Ock Oh, Byoung Soo Kim, Dongjun Lee, Sik Yoon

**Affiliations:** 1 Department of Anatomy, School of Medicine, Pusan National University, Yangsan 50612, Republic of Korea; 2 School of Medicine, Pusan National University, Yangsan 50612, Republic of Korea; 3 Department of Internal Medicine, Pusan National University School of Medicine, Pusan National University Yangsan Hospital, Yangsan 50612, Republic of Korea; 4 Research Institute for Convergence of Biomedical Science and Technology, Pusan National University Yangsan Hospital, Yangsan 50612, Republic of Korea; 5 School of Biomedical Convergence Engineering, Pusan National University, Yangsan 50612, Republic of Korea; 6 Department of Convergence Medicine, Pusan National University College of Medicine, Yangsan 50612, Republic of Korea; 7 Immune Reconstitution Research Center of Medical Research Institute, Pusan National University College of Medicine, Yangsan 50612, Republic of Korea

**Keywords:** marine collagen, scaffold, hydrogel, 3D cell culture, spheroid, hepatocellular carcinoma (HCC), liver cancer stem cells, chemoresistance

## Abstract

The development of physiologically relevant three-dimensional (3D) culture systems is essential for modeling tumor complexity and improving the translational impact of cancer research. We established a 3D in vitro model of human hepatocellular carcinoma (HCC) using a marine collagen peptide-based (MCP-B) biomimetic hydrogel scaffold optimized for multicellular spheroid growth. Compared with conventional two-dimensional (2D) cultures, the MCP-B hydrogel more accurately recapitulated native tumor biology while offering simplicity, reproducibility, bioactivity, and cost efficiency. HCC cells cultured in MCP-B hydrogel displayed tumor-associated behaviors, including enhanced proliferation, colony formation, migration, invasion, and chemoresistance, and enriched cancer stem cell (CSC) populations. Molecular analyses revealed upregulated expression of genes associated with multidrug resistance; stemness regulation and markers; epithelial–mesenchymal transition (EMT) transcription factors, markers, and effectors; growth factors and their receptors; and cancer progression. The spheroids also retained liver-specific functions, suppressed apoptotic signaling, and exhibited extracellular matrix remodeling signatures. Collectively, these findings demonstrate that the 3D HCC model using MCP-B hydrogel recapitulates key hallmarks of tumor biology and provides a robust, physiologically relevant platform for mechanistic studies of HCC and CSC biology. This model further holds translational value for preclinical drug screening and the development of novel anti-HCC and anti-CSC therapeutics.

## 1. Introduction

Liver cancer (LC) remains one of the most challenging malignancies to treat, with annual new cases projected to exceed one million globally by 2025 [1]. In 2022, an estimated 865,269 new LC cases and approximately 757,000 LC-related deaths were reported, representing 4.3% of all new cancer cases and 7.8% of cancer-related deaths [2]. LC ranks as the sixth most commonly diagnosed cancer and the third leading cause of cancer mortality worldwide; among men, it is the fifth most frequent cancer and the second leading cause of cancer death [2,3]. Hepatocellular carcinoma (HCC), which accounts for about 90% of LC cases, is the predominant form and is a major global public health concern [4].

Nearly 90% of HCC cases have an identifiable underlying cause, most commonly chronic viral hepatitis, excessive alcohol consumption, or metabolic dysfunction-associated steatotic liver disease (MASLD) [5]. MASLD, formerly termed non-alcoholic fatty liver disease (NAFLD), is the most prevalent liver disorder globally, with an estimated global prevalence of 38% [6,7]. It includes a spectrum of conditions ranging from simple hepatic steatosis to metabolic dysfunction-associated steatohepatitis (MASH), the progressive and clinically significant form [8]. Hepatitis B virus (HBV) remains the leading cause of HCC, accounting for approximately half of all cases [9]. However, with the implementation of antiviral therapies and vaccination programs, the incidence of HBV- and hepatitis C virus (HCV)-related HCC is steadily declining [10]. In contrast, MASH—previously known as non-alcoholic steatohepatitis (NASH) and is strongly associated with obesity, type 2 diabetes, and insulin resistance—has rapidly emerged as a major and growing cause of HCC worldwide [11,12]. Given the high incidence and poor prognosis of HCC, research in this field remains a critical global health priority.

Primary prevention remains the most effective strategy for reducing HCC-related mortality, and surveillance programs improve outcomes by enabling early detection when curative treatments are still viable. However, despite guideline recommendations, most HCC cases are diagnosed at advanced stages. This delay reflects multiple barriers, including the limited sensitivity of ultrasound-based surveillance (particularly for early-stage tumors), underutilization in clinical practice, inconsistent implementation by clinicians, poor patient adherence, low public awareness, and restricted access to diagnostic services [13,14].

Current systemic therapies for HCC primarily target molecular pathways or the immune system [15]. First-line options include sorafenib, lenvatinib, atezolizumab plus bevacizumab, and tremelimumab plus durvalumab, whereas second-line agents comprise regorafenib, cabozantinib, and ramucirumab [16,17]. Sorafenib, the first approved systemic agent for advanced HCC, provides only a modest survival benefit, with resistance typically arising within six months and benefiting approximately 30% of patients [18,19]. Cabozantinib recently achieved a significant milestone in a global Phase III trial for advanced HCC, meeting its primary endpoint by demonstrating a statistically significant and clinically meaningful improvement in median overall survival compared with placebo [20]. Despite these advances, the prognosis for advanced HCC remains poor, highlighting the urgent need for novel strategies to enhance efficacy and overcome treatment resistance.

Traditional two-dimensional (2D) monolayer cultures have long supported preclinical cancer research, but their limitations are increasingly evident, particularly for modeling complex malignancies such as HCC. A major drawback is their inability to replicate the three-dimensional (3D) architecture, cell–cell and cell–extracellular matrix (ECM) interactions, and microenvironmental gradients present in vivo [21,22,23]. The artificial nature of 2D culture alters key cellular features—including morphology, cytoskeletal organization, gene and protein expression, and intercellular communication—resulting in marked differences in proliferation, differentiation, metabolism, viability, and drug sensitivity [24,25,26,27,28,29]. Therefore, 2D systems fail to capture the genetic and phenotypic heterogeneity of patient tumors and often generate drug response data with limited clinical translatability.

Biomimetic 3D in vitro culture systems address many of these shortcomings by enabling cells to grow and interact with the ECM in all spatial directions, thereby more accurately reproducing tumor structure, function, and microenvironmental conditions [24]. Among these, multicellular spheroid models have gained particular prominence because of their relative simplicity, cost-effectiveness, reproducibility, and capacity to mimic key tumor microenvironmental features such as hypoxic gradients, nutrient diffusion limitations, and cell–cell interactions [30,31,32,33]. Spheroids can be rapidly established from HCC cell lines or patient-derived tumor cells without requiring complex extracellular matrices, making them well suited to high-throughput drug screening and mechanistic studies [30,31,33]. In contrast, organoid models, while valuable for preserving patient-specific histoarchitecture and genetic profiles, often require specialized and costly culture conditions, including growth factor-rich matrices, and may involve longer establishment times, lower throughput capacity, and inconsistent success rates [32,33,34]. Moreover, organoid cultures can be biased toward certain cellular subpopulations, potentially limiting their representation of the full tumor microenvironment [32,33]. Consequently, although organoids remain invaluable for personalized medicine applications, spheroid models currently provide a more practical and scalable platform for preclinical HCC research—particularly in drug efficacy testing, nanomedicine evaluation, and studies of tumor biology under controlled microenvironmental conditions [30,31,32,33,35,36,37,38,39,40].

Recent advances have expanded the use of spheroid-based systems in HCC modeling. Tang et al. (2011) developed a 3D metastatic HCC model using MHCC97H cells cultured on molecular scaffolds, reproducing multiple in vivo tumor characteristics, including morphology, ultrastructure, gene expression, apoptosis, glucose metabolism, and protein biosynthesis [41]. Yip and Cho (2013) established a multicellular hetero-spheroid system embedded in collagen hydrogel to investigate the influence of the tumor microenvironment, cellular heterogeneity, and ECM barriers on drug resistance, yielding important insights into therapy response mechanisms [42]. Terashima et al. (2015) used 3D spheroids of JHH1, Huh7, and HepG2 cells to assess hepatic drug metabolism, observing significantly higher CYP1A1 and CYP1A2 expression than 2D cultures and identifying pregnane X receptor as a key regulator of CYP1A2 upregulation in the 3D setting [43]. These findings highlight the ability of spheroid models to reproduce critical aspects of HCC biology and their growing role as versatile tools for translational cancer research.

However, despite extensive work on 3D in vitro models of HCC, further refinements are required to optimize their efficiency and effectiveness for preclinical applications. In this study, we established a robust 3D in vitro human HCC spheroid model using a biomimetic marine collagen peptide-based (MCP-B) hydrogel matrix specifically engineered for bioactivity, ease of use, and practical applicability. The model incorporates widely used HCC cell lines, including HLF, Huh7, HepG2, PLC/PRF/5, SNU449, and SNU475, which exhibit enhanced malignant phenotypes compared with conventional 2D cultures. These features render the system highly relevant for translational oncology research and anti-cancer drug screening. The structural characteristics and functional performance of the 3D HCC spheroid model using MCP-B hydrogel were comprehensively assessed to validate its suitability for preclinical applications.

## 2. Results

### 2.1. Formation and Growth of HCC Cell Spheroids Were Promoted in MCP-B Hydrogels

**Figure 1:**
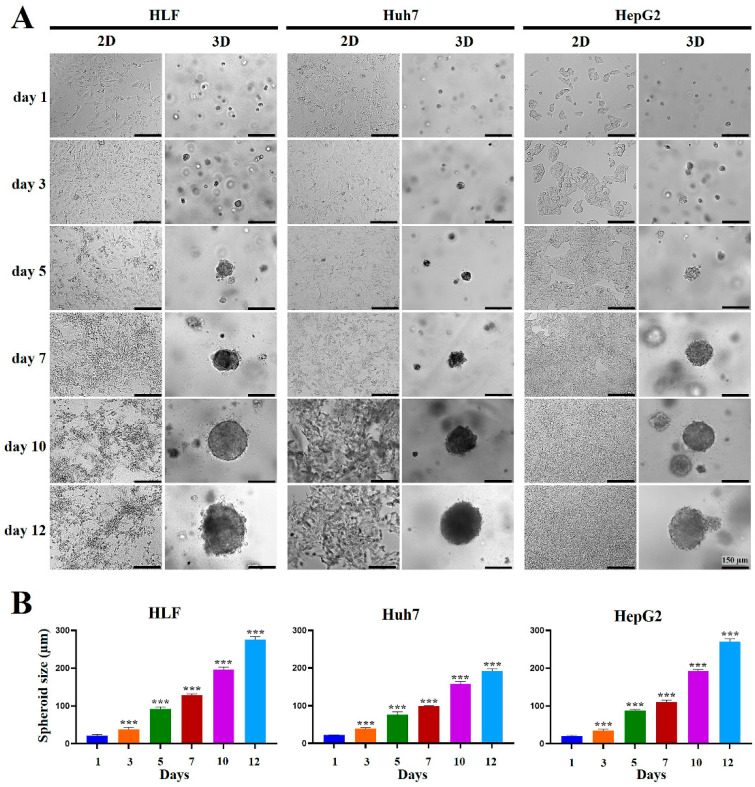
Formation and growth of HCC cell spheroids in standard plastic tissue culture plates and MCP-B hydrogels. (**A**) Phase-contrast microscopy images of HCC cell spheroids (HLF, Huh7, and HepG2) on culture days 1, 3, 5, 7, 10, and 12 (original magnification, ×200). (**B**) Growth of HCC spheroids over time. The average spheroid diameters on days 1, 3, 5, 7, 10, and 12 were 20.8, 37.7, 91.7, 128.7, 196.1, and 275.8 µm, respectively, for HLF cells; 22.2, 39.1, 76.5, 98.8, 157.9, and 191.9 µm, respectively, for Huh7 cells; and 19.8, 34.4, 88.0, 110.1, 192.3, and 270.9, respectively, for HepG2 cells. Data are shown as mean ± standard deviation at each time point from three independent experiments. *** *p* < 0.001 vs. day 1. Scale bars = 150 μm.

Figure 1A illustrates how HCC cells behaved under 2D and 3D conditions over time. When cultured in MCP-B hydrogels, all three cell lines (HLF, Huh7, and HepG2) started forming multiple spheroids within three days (Figure 1A). As culture progressed, both the number and size of spheroids steadily increased (Figure 1A,B). Although the growth rate varied slightly among cell types (HLF ≈ HepG2 > Huh7), their spheroids followed a similar overall growth pattern (Figure 1A,B). By day 12, nearly all spheroids exceeded 190 µm in diameter (Figure 1A,B), indicating that MCP-B hydrogels provide a supportive environment for spheroid formation and expansion. This system may therefore serve as a useful platform for studying HCC growth dynamics and for exploratory applications in experimental testing.

### 2.2. Proliferation and Clonogenicity of HCC Cells Were Enhanced in MCP-B Hydrogels

**Figure 2:**
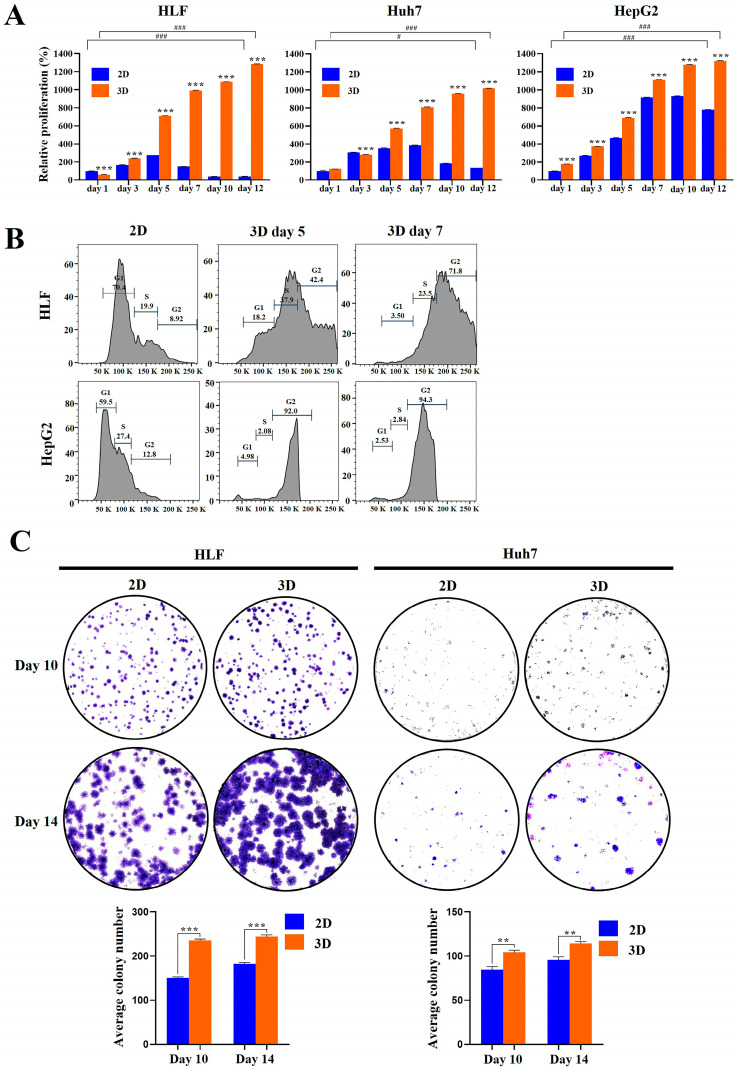
Proliferation of HCC cells in standard plastic tissue culture plates and MCP-B hydrogels. (**A**) WST-1 assay of HLF, Huh7, and HepG2 cells cultured in 2D (day 1) and in 3D (days 1, 3, 5, 7, 10, and 12). (**B**) HLF and HepG2 cells in 3D culture showed a reduced proportion in the G0/G1 phase and an increased proportion in the S and G2/M phases compared with 2D, indicating enhanced proliferation. (**C**) Colony formation assay of HLF and Huh7 cells cultured under 2D and 3D conditions for 10 and 14 days. Data are presented as mean ± standard deviation of three independent experiments. ** *p* < 0.01, *** *p* < 0.001 vs. 2D. ^#^ *p* < 0.05, ^###^ *p* < 0.001 vs. those on day 1.

A water-soluble tetrazolium (WST)-1 colorimetric assay was used to quantify the ability of MCP-B hydrogels to promote cell proliferation. HCC cells were successfully propagated in the MCP-B hydrogels. After 5 days of culture, cell numbers in 3D cultures exceeded those in 2D cultures for all three cell types (Figure 2A). On days 5, 7, 10, and 12, the proliferation rates in 3D compared with 2D cultures increased 2.6-fold (*p* < 0.001), 6.7-fold (*p* < 0.001), 26.6-fold (*p* < 0.001), and 33.8-fold (*p* < 0.001), respectively, for HLF cells; 1.6-fold (*p* < 0.001), 2.1-fold (*p* < 0.001), 5.2-fold (*p* < 0.001), and 7.4-fold (*p* < 0.001), respectively, for Huh7 cells; and 1.5-fold (*p* < 0.001), 1.2-fold (*p* < 0.001), 1.4-fold (*p* < 0.001), and 1.7-fold (*p* < 0.001), respectively, for HepG2 cells (Figure 2A).

Cell cycle progression is a key regulator of cancer cell proliferation. Tumor cells cultured in 3D systems, which better mimic the physiological microenvironment, are often reported to display altered cell cycle dynamics compared with those grown in conventional 2D monolayers [44]. Based on this, we hypothesized that 3D spheroids would exhibit distinct cell cycle profiles relative to their 2D counterparts. To evaluate this, cell cycle distribution was analyzed in HLF and HepG2 cells using propidium iodide (PI) staining followed by flow cytometry. Both HLF and HepG2 cells cultured under 3D conditions exhibited markedly altered cell cycle distributions. For HLF cells, the proportion of cells in the G0/G1 phase was 18.2% (day 5, *p* < 0.001) and 3.5% (day 7, *p* < 0.001) in 3D cultures compared with 70.4% in 2D. In contrast, the fraction in the S/G2/M phases reached 80.3% (day 5, *p* < 0.001) and 95.3% (day 7, *p* < 0.001) in 3D compared with 28.8% in 2D (Figure 2B). Similarly, for HepG2 cells, the proportion in the G0/G1 phase was 5.0% (day 5, *p* < 0.001) and 2.5% (day 7, *p* < 0.001) in 3D compared with 59.5% in 2D, whereas the S/G2/M fraction was 94.1% (day 5, *p* < 0.001) and 97.1% (day 7, *p* < 0.001) in 3D compared with 40.2% in 2D (Figure 2B).

The G2/M phase in 3D-cultured cells showed a significantly higher percentage than 2D-cultured cells in both HepG2 and HLF lines. These results suggest that cells cultured in 3D MCP-B hydrogel exhibit a more active cell cycle profile than in 2D, characterized by a reduced proportion in the G0/G1 phase and an increased proportion in the S and G2/M phases. These findings indicate that 3D cell culture in MCP-B hydrogel promotes cell cycle progression, thereby enhancing proliferative activity and supporting a growth-favorable tumor microenvironment compared with 2D culture.

To evaluate colony-forming ability in MCP-B hydrogels, a clonogenicity assay was performed. For HLF cells, the average number of colonies formed under 3D culture conditions was significantly higher than under 2D conditions. At day 10, 3D culture yielded 235.3 ± 3.05 colonies compared with 150.3 ± 2.52 colonies in 2D (a 1.6-fold increase, *p* < 0.001) (Figure 2C). At day 14, the average colony number was 244.3 ± 4.04 in 3D vs. 182.3 ± 3.05 in 2D (a 1.3-fold increase, *p* < 0.001) (Figure 2C). Similarly, for Huh7 cells, the average colony number under 3D conditions was 104.3 ± 2.52 compared with 84.6 ± 3.51 in 2D at day 10 (a 1.2-fold increase, *p* < 0.01), and 114.3 ± 2.08 in 3D vs. 95.6 ± 3.51 in 2D at day 14 (a 1.2-fold increase, *p* < 0.01) (Figure 2C). These results confirm that MCP-B hydrogels promote greater clonogenic potential compared with 2D culture. Collectively, these findings, together with the results of the cell proliferation assay, indicate that MCP-B hydrogels produce conditions more favorable for HCC cell proliferation and colony formation than 2D culture.

### 2.3. Metastatic Potential of HCC Cells Was Elevated in MCP-B Hydrogels

Metastasis is a multistep process involving the migration and invasion of cancer cells, which are hallmark features of cancer progression [45,46,47]. Tumor cells grown in 3D models, which more accurately recapitulate the complexity of in vivo biological systems, are considered to exhibit higher metastatic potential than those cultured in traditional 2D monolayers. Therefore, we hypothesized that 3D multicellular HCC spheroids would display enhanced metastatic potential compared with cells cultured in 2D. To assess this, wound-healing and invasion assays were performed using HLF and Huh7 cells.

**Figure 3:**
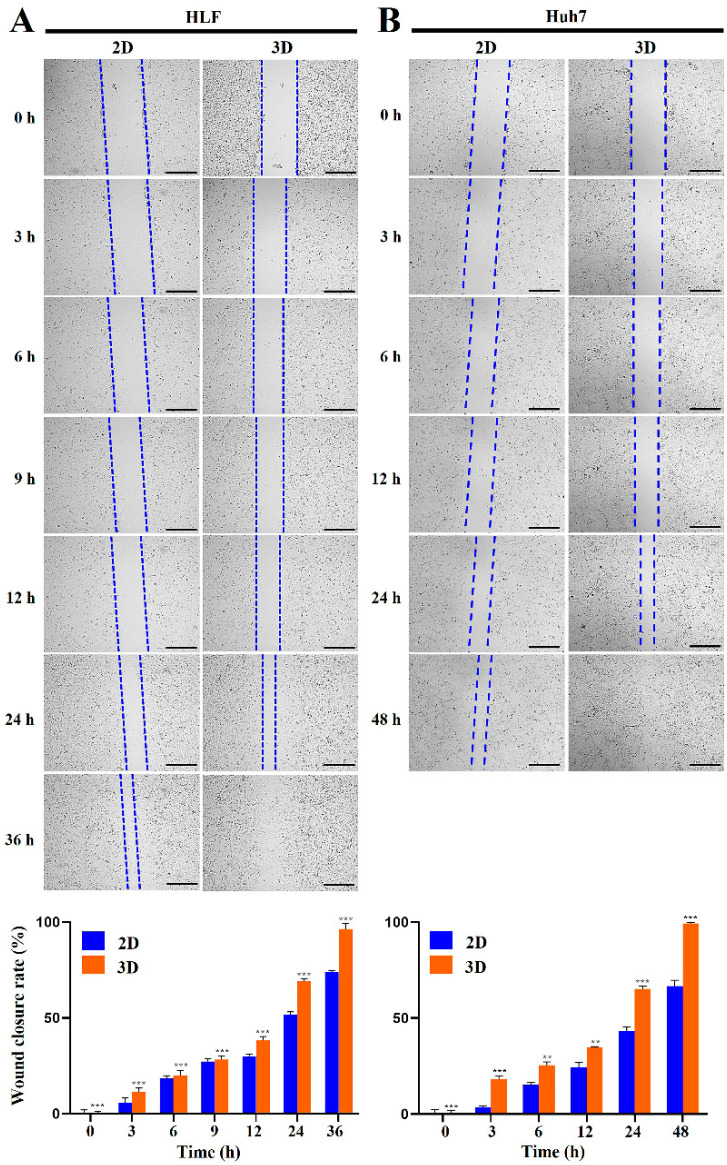
Migratory behavior of HCC cancer cells in standard plastic tissue culture plates and MCP-B hydrogels. Representative phase-contrast microscopy images from a wound-healing assay of HLF and Huh7 cells. Images were captured at 0, 3, 6, 9, 12, 24, and 36 h (original magnification, ×40) for HLF (**A**) cells and 0, 3, 6, 12, 24, and 48 h (original magnification, ×40) for Huh7 (**B**) cells. Distances between the two edges were measured at three positions across different time points. Data are presented as the mean ± standard deviation of three independent experiments. ** *p* < 0.01 and *** *p* < 0.001 vs. 2D. Scale bars = 650 μm.

For HLF cells, images of scratch areas in the wound-healing assay were obtained at 3, 6, 9, 12, 24, and 36 h. The rate of wound closure in 3D cultures was 11.4% (vs. 5.7% in 2D, *p* < 0.001), 19.9% (vs. 18.4% in 2D, *p* < 0.001), 28.3% (vs. 27.1% in 2D, *p* < 0.001), 38.2% (vs. 29.9% in 2D, *p* < 0.001), 69.1% (vs. 51.8% in 2D, *p* < 0.001), and 96.4% (vs. 74.0% in 2D, *p* < 0.001) at 3, 6, 9, 12, 24, and 36 h, respectively (Figure 3). For Huh7 cells, images of scratch areas were obtained at 3, 6, 12, 24, and 48 h. The rate of wound closure in 3D cultures was 18.2% (vs. 3.5% for 2D, *p* < 0.001), 25.5% (vs. 15.1% in 2D, *p* < 0.01), 34.6% (vs. 24.4% in 2D, *p* < 0.01), 65.1% (vs. 43.3% in 2D, *p* < 0.001), and 99.3% (vs. 66.6% in 2D, *p* < 0.001) at 3, 6, 12, 24, and 48 h, respectively (Figure 3).

**Figure 4:**
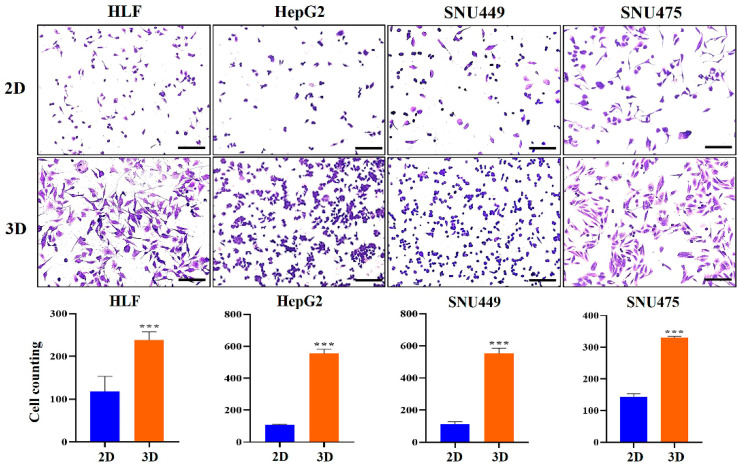
Invasive behavior of HCC cells in standard plastic tissue culture plates and marine collagen-based hydrogels. Representative images of the hydrogel invasion assay with HCC cells. 3D-cultured HCC cells (HLF, HepG2, SNU449, and SNU475) showed significantly increased invasiveness compared with 2D-cultured cells (original magnification, ×100). Data are presented as mean ± SD of three independent experiments. *** *p* < 0.001 vs. respective 2D control. Scale bars: 275 μm.

Additionally, hydrogel invasion assays showed that 3D-cultured cells exhibited a significantly greater number of invasive cells compared with 2D-cultured cells (Figure 4). The invasion capacity of the 3D-cultured group was significantly higher than that of the 2D control group at 18 h in HCC cells. Specifically, for HLF cells, the number of invading cells under 3D culture conditions (mean ± standard deviation [SD]: 238.8 ± 19.09) was approximately 2.0-fold higher (*p* < 0.01) than that under 2D conditions (118.3 ± 35.13) (Figure 4). For HepG2 cells, the number of invading cells in 3D culture (554.0 ± 28.16) was approximately 5.1-fold higher (*p* < 0.001) than in 2D culture (108.3 ± 4.16) (Figure 4). In SNU449 cells, the number of invading cells in 3D culture (552.0 ± 33.50) was approximately 4.9-fold higher (*p* < 0.001) than in 2D culture (112.8 ± 14.24) (Figure 4). For SNU475 cells, the number of invading cells in 3D culture (330.3 ± 4.04) was approximately 2.1-fold higher (*p* < 0.001) than in 2D culture (156.3 ± 7.02) (Figure 4). Collectively, these findings demonstrate a significant overall increase in the invasiveness of HCC cells in 3D culture in MCP-B hydrogels compared with 2D monolayers (Figure 4).

### 2.4. Chemoresistance of HCC Cells Was Increased in MCP-B Hydrogels

**Figure 5:**
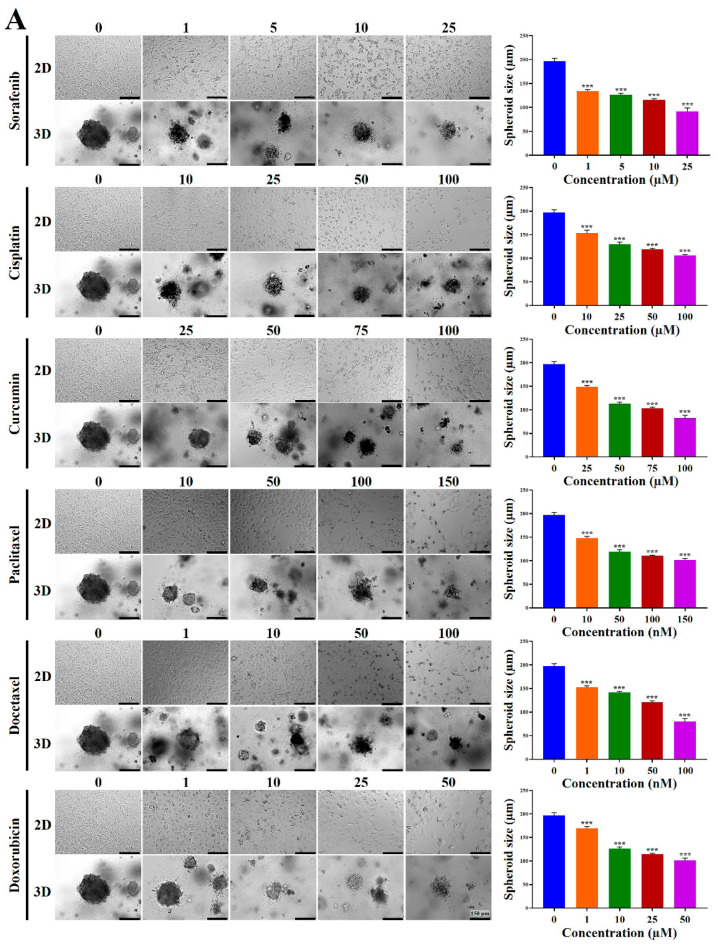

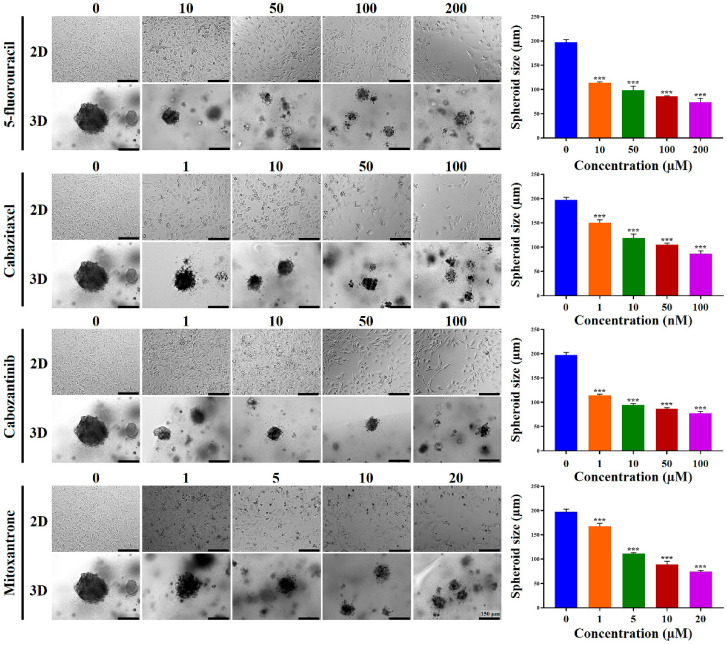

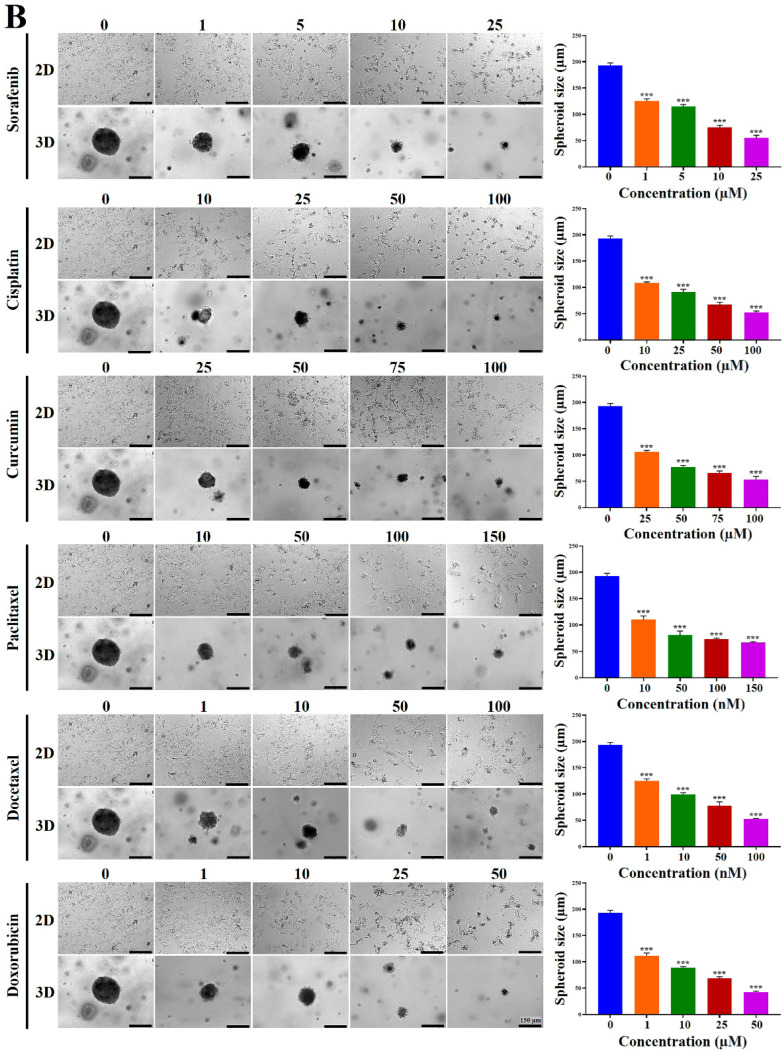

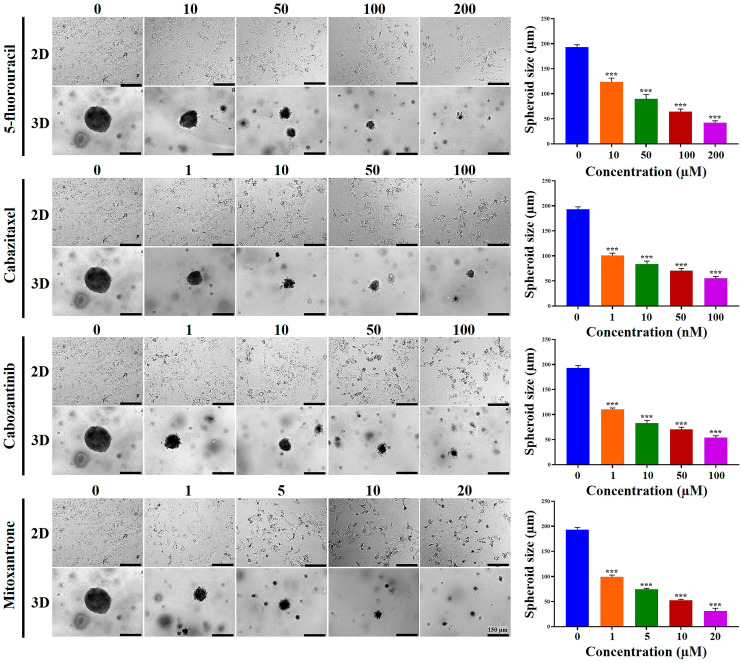

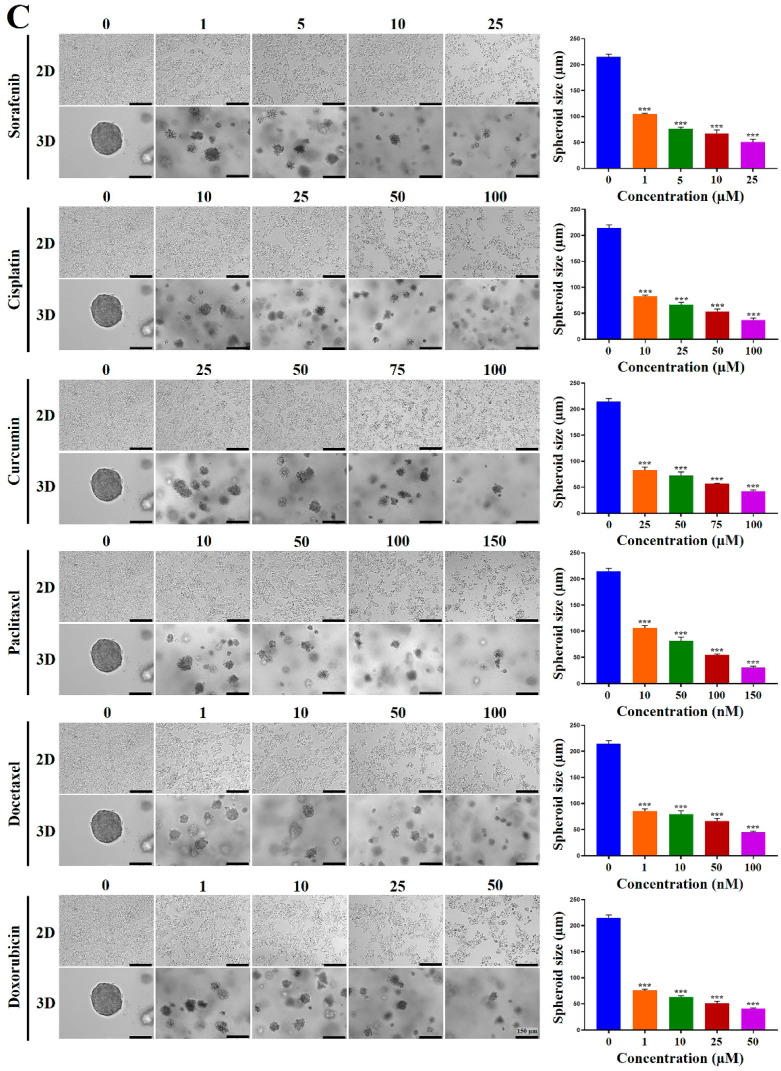

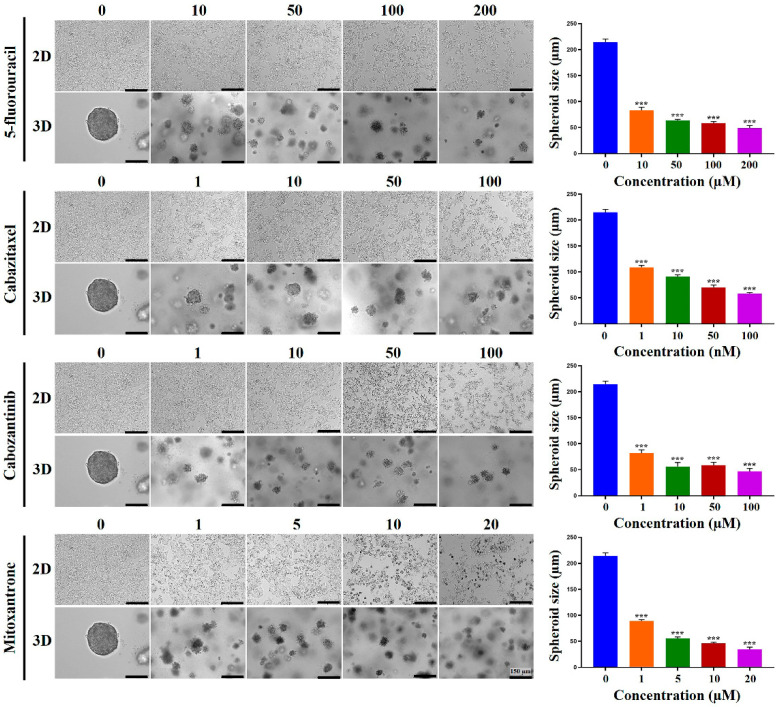
Chemoresistance of 2D- and 3D-cultured HCC cells in standard plastic tissue culture plates and MCP-B hydrogels. (**A**) Morphology and spheroid size of HLF cells at 24 h after exposure to sorafenib, cisplatin, curcumin, paclitaxel, docetaxel, doxorubicin, 5-fluorouracil, cabazitaxel, cabozantinib, and mitoxantrone in 2D and 3D culture. (**B**) Morphology and spheroid size of Huh7 cells at 24 h after exposure to sorafenib, cisplatin, curcumin, paclitaxel, docetaxel, doxorubicin, 5-fluorouracil, cabazitaxel, cabozantinib, and mitoxantrone in 2D and 3D culture. (**C**) Morphology and spheroid size of HepG2 cells at 24 h after exposure to sorafenib, cisplatin, curcumin, paclitaxel, docetaxel, doxorubicin, 5-fluorouracil, cabazitaxel, cabozantinib, and mitoxantrone in 2D and 3D culture. Data are presented as mean spheroid size ± standard deviation from three independent experiments, normalized to untreated controls. *** *p* < 0.001 vs. 3D control. Scale bars = 150 μm.

Multidrug resistance remains a major obstacle to successful cancer chemotherapy. Tumor cells grown in 3D models, which more accurately mimic the properties of living tissues, typically exhibit higher levels of drug resistance than those cultured in traditional 2D monolayers. Thus, we hypothesized that 3D multicellular HCC spheroids grown within MCP-B hydrogels would display enhanced resistance to anti-cancer agents compared with 2D-cultured cells. HLF, Huh7, and HepG2 cells were cultured for 24 h in the presence of serial concentrations of sorafenib, cisplatin, curcumin, paclitaxel, docetaxel, doxorubicin, 5-fluorouracil, cabazitaxel, cabozantinib, and mitoxantrone under both 2D and 3D conditions. To evaluate the effects of the 3D microenvironment provided by MCP-B hydrogels on drug resistance, cell viability was assessed using a WST-1 colorimetric assay. These cytotoxicity results were consistent with morphological changes observed by phase-contrast microscopy (Figure 5).

First, we assessed cellular cytotoxicity in 2D- and 3D-cultured HCC cells after treatment with various chemotherapeutic agents. In 3D-cultured HLF cells, all tested agents induced a dose-dependent reduction in spheroid diameters after 24 h of treatment (Figure 5A). At baseline, spheroid diameter was 197.0 μm. Sorafenib reduced diameters to 133.5, 125.9, 115.6, and 91.6 μm at 1, 5, 10, and 25 μM, respectively. Cisplatin reduced diameters to 153.3, 129.8, 119.0, and 106.1 μm at 10, 25, 50, and 100 μM, respectively. Curcumin reduced diameters to 148.7, 112.8, 103.4, and 82.5 μm at 25, 50, 75, and 100 μM, respectively. Paclitaxel reduced diameters to 148.1, 119.6, 110.4, and 101.8 μm at 10, 50, 100, and 150 nM, respectively. Docetaxel reduced diameters to 152.1, 141.1, 121.0, and 80.3 μm at 1, 10, 50, and 100 nM, respectively. Doxorubicin reduced diameters to 169.6, 125.9, 114.7, and 101.0 μm at 1, 10, 25, and 50 μM, respectively. 5-fluorouracil reduced diameters to 113.6, 97.8, 85.7, and 73.5 μm at 10, 50, 100, and 200 μM, respectively. Cabazitaxel reduced diameters to 150.1, 118.9, 104.9, and 86.2 μm at 1, 10, 50, and 100 nM, respectively. Cabozantinib reduced diameters to 114.5, 94.3, 87.2, and 77.7 μm at 1, 10, 50, and 100 μM, respectively. Finally, mitoxantrone also reduced diameters to 167.3, 111.2, 88.9, and 74.5 μm at 1, 5, 10, and 20 μM, respectively.

Similarly, in 3D-cultured Huh7 cells, all tested agents induced a dose-dependent reduction in spheroid diameters after 24 h of treatment (Figure 5B). At baseline, spheroid diameter was 193.3 μm. Sorafenib reduced diameters to 125.7, 114.6, 75.6, and 55.0 μm at 1, 5, 10, and 25 μM, respectively. Cisplatin reduced diameters to 108.1, 90.8, 67.4, and 52.3 μm at 10, 25, 50, and 100 μM, respectively. Curcumin reduced diameters to 105.8, 76.9, 65.4, and 53.1 μm at 25, 50, 75, and 100 μM, respectively. Paclitaxel reduced diameters to 110.1, 81.7, 73.1, and 66.6 μm at 10, 50, 100, and 150 nM, respectively. Docetaxel reduced diameters to 124.7, 99.4, 77.1, and 52.2 μm at 1, 10, 50, and 100 nM, respectively. Doxorubicin reduced diameters to 111.1, 88.5, 68.4, and 42.4 μm at 1, 10, 25, and 50 μM, respectively. 5-fluorouracil reduced diameters to 123.8, 89.8, 64.4, and 42.3 μm at 10, 50, 100, and 200 μM, respectively. Cabazitaxel reduced diameters to 100.6, 83.8, 70.8, and 55.2 μm at 1, 10, 50, and 100 nM, respectively. Cabozantinib reduced diameters to 110.7, 82.7, 70.3, and 53.8 μm at 1, 10, 50, and 100 μM, respectively. Finally, mitoxantrone also reduced diameters to 99.6, 74.7, 52.9, and 31.3 μm at 1, 5, 10, and 20 μM, respectively.

Consistently, in 3D-cultured HepG2 cells, all tested agents induced a dose-dependent reduction in spheroid diameters after 24 h of treatment (Figure 5C). At baseline, spheroid diameter was 214.8 μm. Sorafenib reduced diameters to 104.9, 76.6, 67.8, and 50.7 μm at 1, 5, 10, and 25 μM, respectively. Cisplatin reduced diameters to 82.4, 67.1, 53.9, and 36.8 μm at 10, 25, 50, and 100 μM, respectively. Curcumin reduced diameters to 83.1, 72.5, 56.8, and 42.5 μm at 25, 50, 75, and 100 μM, respectively. Paclitaxel reduced diameters to 106.1, 81.2, 54.6, and 30.2 μm at 10, 50, 100, and 150 nM, respectively. Docetaxel reduced diameters to 85.7, 79.6, 66.5, and 44.6 μm at 1, 10, 50, and 100 nM, respectively. Doxorubicin reduced diameters to 75.7, 62.9, 51.3, and 40.7 μm at 1, 10, 25, and 50 μM, respectively. 5-fluorouracil reduced diameters to 83.2, 63.3, 58.5, and 49.4 μm at 10, 50, 100, and 200 μM, respectively. Cabazitaxel reduced diameters to 108.9, 90.9, 70.3, and 58.6 μm at 1, 10, 50, and 100 nM, respectively. Cabozantinib reduced diameters to 81.8, 56.3, 58.8, and 46.7 μm at 1, 10, 50, and 100 μM, respectively. Finally, mitoxantrone also reduced diameters to 89.2, 55.1, 46.5, and 34.5 μm at 1, 5, 10, and 20 μM, respectively.

**Figure 6:**
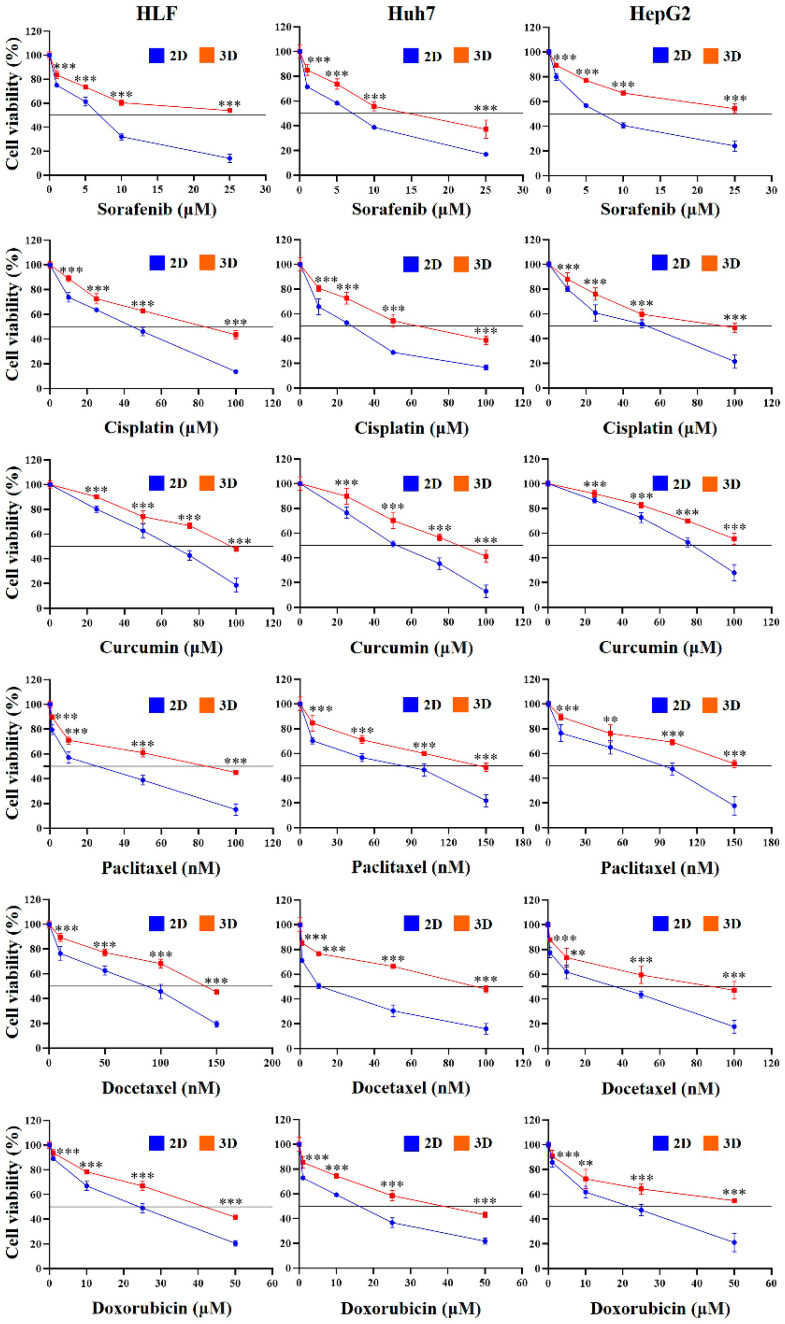

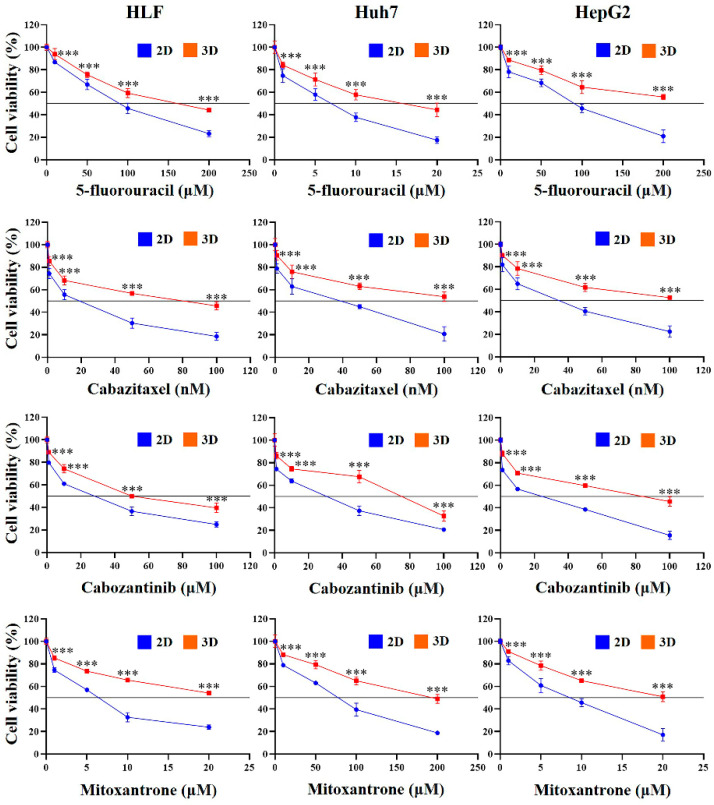
Chemoresistance of 2D- and 3D-cultured HCC cells in standard plastic tissue culture plates and MCP-B hydrogels. Cell viability of HLF, Huh7, and HepG2 cells at 24 h after treatment with sorafenib, cisplatin, curcumin, paclitaxel, docetaxel, doxorubicin, 5-fluorouracil, cabazitaxel, cabozantinib, and mitoxantrone in 2D and 3D cultures. Data are presented as mean percentage viability ± standard deviation of three independent experiments, normalized to untreated controls. ** *p* < 0.01 and *** *p* < 0.001 vs. 2D.

Next, we assessed cell viability in 2D- and 3D-cultured HLF, Huh7, and HepG2 cells after treatment with various chemotherapeutic agents. Across all three cell lines, 3D cultures exhibited a dose-dependent increase in cell viability after 24 h compared with 2D cultures, indicating enhanced chemoresistance (Figure 6). In 3D-cultured HLF cells, sorafenib increased viability 1.1-fold (*p* < 0.001), 1.2-fold (*p* < 0.001), 1.9-fold (*p* < 0.001), and 3.8-fold (*p* < 0.001) at 1, 5, 10, and 25 μM, respectively, compared with 2D-cultured cells. Cisplatin increased viability 1.2-fold (*p* < 0.001), 1.1-fold (*p* < 0.001), 1.4-fold (*p* < 0.001), and 3.2-fold (*p* < 0.001) at 10, 25, 50, and 100 μM, respectively. Curcumin increased viability 1.1-fold (*p* < 0.001), 1.2-fold (*p* < 0.001), 1.6-fold (*p* < 0.001), and 2.6-fold (*p* < 0.001) at 25, 50, 75, and 100 μM, respectively. Paclitaxel increased viability 1.2-fold (*p* < 0.001), 1.2-fold (*p* < 0.001), 1.5-fold (*p* < 0.001), and 2.3-fold (*p* < 0.001) at 10, 50, 100, and 150 nM, respectively.

Docetaxel increased viability 1.1-fold (*p* < 0.001), 1.2-fold (*p* < 0.001), 1.6-fold (*p* < 0.001), and 3.0-fold (*p* < 0.001) at 1, 10, 50, and 100 nM, respectively. Doxorubicin increased viability 1.1-fold (*p* < 0.001), 1.2-fold (*p* < 0.001), 1.4-fold (*p* < 0.001), and 2.0-fold (*p* < 0.001) at 1, 10, 25, and 50 μM, respectively. 5-fluorouracil increased viability 1.1-fold (*p* < 0.001), 1.1-fold (*p* < 0.001), 1.3-fold (*p* < 0.001), and 1.9-fold (*p* < 0.001) at 10, 50, 100, and 200 μM, respectively. Cabazitaxel increased viability 1.1-fold (*p* < 0.001), 1.2-fold (*p* < 0.001), 1.9-fold (*p* < 0.001), and 2.5-fold (*p* < 0.001) at 1, 10, 50, and 100 nM, respectively. Cabozantinib increased viability 1.1-fold (*p* < 0.001), 1.2-fold (*p* < 0.001), 1.4-fold (*p* < 0.001), and 1.6-fold (*p* < 0.001) at 1, 10, 50, and 100 μM, respectively. Mitoxantrone increased viability 1.1-fold (*p* < 0.001), 1.3-fold (*p* < 0.001), 2.0-fold (*p* < 0.001), and 2.3-fold (*p* < 0.001) at 1, 5, 10, and 20 μM, respectively.

Similarly, in 3D-cultured Huh7 cells, all tested agents produced a dose-dependent increase in cell viability after 24 h (Figure 6). Sorafenib increased viability 1.2-fold (*p* < 0.001), 1.3-fold (*p* < 0.001), 1.4-fold (*p* < 0.001), and 2.2-fold (*p* < 0.001) at 1, 5, 10, and 25 μM, respectively. Cisplatin increased viability 1.2-fold (*p* < 0.001), 1.4-fold (*p* < 0.001), 1.9-fold (*p* < 0.001), and 2.3-fold (*p* < 0.001) at 10, 25, 50, and 100 μM, respectively. Curcumin increased viability 1.2-fold (*p* < 0.001), 1.4-fold (*p* < 0.001), 1.6-fold (*p* < 0.001), and 3.2-fold (*p* < 0.001) at 25, 50, 75, and 100 μM, respectively. Paclitaxel increased viability 1.2-fold (*p* < 0.001), 1.3-fold (*p* < 0.001), 1.3-fold (*p* < 0.001), and 2.2-fold (*p* < 0.001) at 10, 50, 100, and 150 nM, respectively. Docetaxel increased viability 1.2-fold (*p* < 0.001), 1.5-fold (*p* < 0.001), 2.2-fold (*p* < 0.001), and 3.0-fold (*p* < 0.001) at 1, 10, 50, and 100 nM, respectively. Doxorubicin increased viability 1.2-fold (*p* < 0.001), 1.3-fold (*p* < 0.001), 1.6-fold (*p* < 0.001), and 2.0-fold (*p* < 0.001) at 1, 10, 25, and 50 μM, respectively. 5-fluorouracil increased viability 1.1-fold (*p* < 0.001), 1.3-fold (*p* < 0.001), 1.6-fold (*p* < 0.001), and 2.6-fold (*p* < 0.001) at 10, 50, 100, and 200 μM, respectively.

Cabazitaxel increased viability 1.1-fold (*p* < 0.001), 1.2-fold (*p* < 0.001), 1.4-fold (*p* < 0.001), and 2.6-fold (*p* < 0.001) at 1, 10, 50, and 100 nM, respectively. Cabozantinib increased viability 1.2-fold (*p* < 0.001), 1.2-fold (*p* < 0.001), 1.8-fold (*p* < 0.001), and 1.6-fold (*p* < 0.001) at 1, 10, 50, and 100 μM, respectively. Mitoxantrone also increased viability 1.1-fold (*p* < 0.001), 1.2-fold (*p* < 0.001), 1.5-fold (*p* < 0.001), and 2.5-fold (*p* < 0.001) at 1, 5, 10, and 20 μM, respectively.

Consistently, in 3D-cultured HepG2 cells, all tested agents produced a dose-dependent increase in cell viability after 24 h (Figure 6). Sorafenib increased viability 1.1-fold (*p* < 0.001), 1.4-fold (*p* < 0.001), 1.6-fold (*p* < 0.001), and 2.3-fold (*p* < 0.001) at 1, 5, 10, and 25 μM, respectively. Cisplatin increased viability 1.1-fold (*p* < 0.001), 1.3-fold (*p* < 0.001), 1.2-fold (*p* < 0.001), and 2.3-fold (*p* < 0.001) at 10, 25, 50, and 100 μM, respectively. Curcumin increased viability 1.1-fold (*p* < 0.001), 1.1-fold (*p* < 0.001), 1.3-fold (*p* < 0.001), and 2.0-fold (*p* < 0.001) at 25, 50, 75, and 100 μM, respectively.

Paclitaxel increased viability 1.2-fold (*p* < 0.001), 1.2-fold (*p* < 0.001), 1.5-fold (*p* < 0.001), and 2.9-fold (*p* < 0.001) at 10, 50, 100, and 150 nM, respectively. Docetaxel increased viability 1.1-fold (*p* < 0.001), 1.2-fold (*p* < 0.001), 1.4-fold (*p* < 0.001), and 2.7-fold (*p* < 0.001) at 1, 10, 50, and 100 nM, respectively. Doxorubicin increased viability 1.1-fold (*p* < 0.001), 1.2-fold (*p* < 0.001), 1.4-fold (*p* < 0.001), and 2.6-fold (*p* < 0.001) at 1, 10, 25, and 50 μM, respectively. 5-fluorouracil increased viability 1.1-fold (*p* < 0.001), 1.2-fold (*p* < 0.001), 1.4-fold (*p* < 0.001), and 2.7-fold (*p* < 0.001) at 10, 50, 100, and 200 μM, respectively.

Cabazitaxel increased viability 1.1-fold (*p* < 0.001), 1.2-fold (*p* < 0.001), 1.5-fold (*p* < 0.001), and 2.3-fold (*p* < 0.001) at 1, 10, 50, and 100 nM, respectively. Cabozantinib increased viability 1.2-fold (*p* < 0.001), 1.2-fold (*p* < 0.001), 1.5-fold (*p* < 0.001), and 2.9-fold (*p* < 0.001) at 1, 10, 50, and 100 μM, respectively. Mitoxantrone increased viability 1.1-fold (*p* < 0.001), 1.3-fold (*p* < 0.001), 1.4-fold (*p* < 0.001), and 3.0-fold (*p* < 0.001) at 1, 5, 10, and 20 μM, respectively. Collectively, these results demonstrate that MCP-B hydrogels confer enhanced chemoresistance across multiple HCC cell lines compared with 2D cultures.

**Figure 7:**
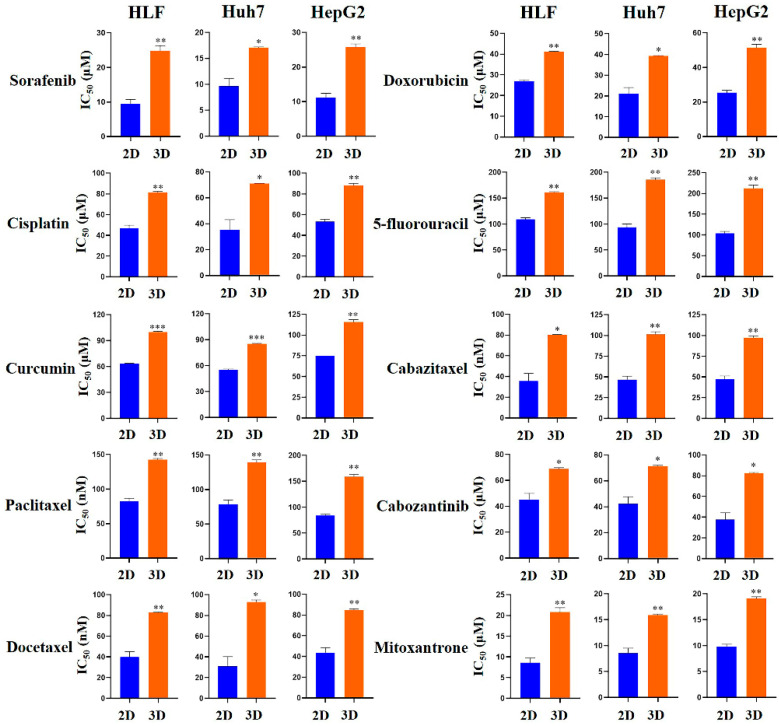
IC_50_ values of HCC cells after 24 h of treatment with sorafenib, cisplatin, curcumin, paclitaxel, docetaxel, doxorubicin, 5-fluorouracil, cabazitaxel, cabozantinib, and mitoxantrone in HLF, Huh7, and HepG2 cells cultured in standard plastic tissue culture plates and MCP-B hydrogels. Data are presented as mean IC_50_ ± standard deviation of three independent experiments, normalized to 2D cultures. * *p* < 0.05, ** *p* < 0.01 and *** *p* < 0.001 vs. 2D.

Furthermore, the half-maximal inhibitory concentration (IC_50_) values of various chemotherapeutic agents were determined in 2D- and 3D-cultured HLF, Huh7, and HepG2 cells after 24 h of treatment. Across all three cell lines, 3D cultures in MCP-B hydrogels consistently exhibited higher IC_50_ values than 2D cultures, reflecting reduced drug sensitivity and enhanced chemoresistance (Figure 7). For HLF cells, sorafenib inhibited 2D-cultured cells with an IC_50_ of 9.4 μM, whereas reduced sensitivity was observed in 3D-cultured cells (24.9 μM). Cisplatin and curcumin were also less effective in 3D (81.6 and 100.1 μM, respectively) than in 2D (47.1 and 63.7 μM, respectively). Paclitaxel (142.7 nM vs. 82.6 nM) and docetaxel (82.7 vs. 39.9 nM), and doxorubicin (41.2 vs. 26.8 μM) likewise showed increased IC_50_ values in 3D than in 2D. Similarly, 5-fluorouracil (161.7 vs. 109.3 μM), cabazitaxel (80.1 vs. 35.5 nM), cabozantinib (68.9 vs. 44.9 μM), and mitoxantrone (20.8 vs. 8.6 μM) were less effective in 3D cultures compared with 2D.

For Huh7 cells, sorafenib showed an IC_50_ of 9.7 μM in 2D cultures vs. 17.1 μM in 3D. Cisplatin and curcumin were less effective in 3D (71.0 and 85.4 μM, respectively) than in 2D (35.2 and 55.7 μM, respectively). Paclitaxel (139.4 vs. 78.8 nM), docetaxel (93.1 vs. 31.0 nM), and doxorubicin (39.3 vs. 21.3 μM) displayed similar patterns. Increased IC_50_ values were also observed in 3D cultures compared with 2D for 5-fluorouracil (185.7 vs. 94.5 μM), cabazitaxel (101.4 vs. 46.7 nM), cabozantinib (71.4 vs. 42.3 μM), and mitoxantrone (15.9 vs. 8.6 μM).

For HepG2 cells, sorafenib exhibited an IC_50_ of 11.2 μM in 2D vs. 25.7 μM in 3D cultures. Cisplatin and curcumin were less effective in 3D (88.3 and 115.7 μM, respectively) than in 2D (53.5 and 75.2 μM, respectively). Paclitaxel (159.2 vs. 83.6 nM), docetaxel (85.1 vs. 43.8 nM), and doxorubicin (51.5 vs. 25.5 μM) also demonstrated increased IC_50_ values in 3D than in 2D. Similarly, 5-fluorouracil (212.6 vs. 103.9 μM), cabazitaxel (97.4 vs. 47.3 nM), cabozantinib (82.5 vs. 37.7 μM), and mitoxantrone (19.1 vs. 9.8 μM) showed reduced efficacy in 3D cultures compared with 2D.

Together, these findings demonstrate that 3D cultures in MCP-B hydrogels exhibit uniformly higher IC_50_ values than 2D monolayers across multiple chemotherapeutic agents, confirming enhanced chemoresistance and highlighting the suitability of this 3D system for drug testing and therapeutic evaluation.

### 2.5. Stemness-Related Marker Expression of HCC Cells Was Enhanced in MCP-B Hydrogels

The expression of key genes associated with cancer stemness and pluripotency was evaluated to determine the cancer stem cell (CSC) enrichment efficiency of the 3D HCC cell culture model using MCP-B hydrogels.

**Figure 8:**
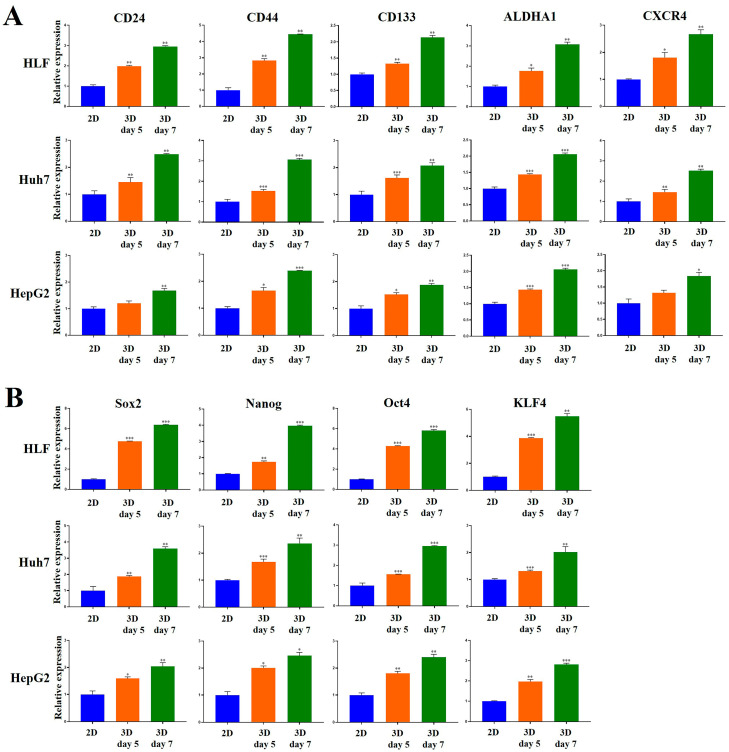
Expression of stemness- and pluripotency-related biomarkers in HCC cell spheroids (days 5 and 7) compared with 2D cultures (day 1). (**A**) Gene expression of CSC markers (CD24, CD44, CD133, ALDHA1, and CXCR4) (**B**) Expression of stemness- and pluripotency-regulating transcription factors (Sox2, Nanog, Oct4, and KLF4). Gene expression was analyzed by qRT-polymerase chain reaction (PCR), with glyceraldehyde 3-phosphate dehydrogenase (GAPDH) used as a housekeeping gene for normalization. Data represent the mean ± standard deviation of three independent experiments. * *p* < 0.05, ** *p* < 0.01, and *** *p* < 0.001 vs. 2D.

First, we examined the expression of typical CSC surface markers for HCC, including CD24, CD44, CD133, aldehyde dehydrogenase 1 family member A1 (ALDHA1), and CXCR4. All five markers were robustly elevated in 3D compared with 2D cultures across HLF, Huh7, and HepG2 cells (Figure 8A). In 3D-cultured HLF cells, expression levels at day 5 were significantly increased by 1.9-fold (CD24, *p* < 0.01), 2.8-fold (CD44, *p* < 0.01), 1.3-fold (CD133, *p* < 0.01), 1.8-fold (ALDHA1, *p* < 0.05), and 1.8-fold (CXCR4, *p* < 0.05), respectively, compared with 2D cultures. By day 7, these increases were more pronounced, reaching 2.9-fold (CD24, *p* < 0.01), 4.5-fold (CD44, *p* < 0.01), 2.1-fold (CD133, *p* < 0.01), 3.1-fold (ALDHA1, *p* < 0.01), and 2.7-fold (CXCR4, *p* < 0.01), respectively.

Three-dimensionally-cultured Huh7 cells, at day 5, exhibited 1.5-fold (CD24, *p* < 0.01), 1.5-fold (CD44, *p* < 0.001), 1.6-fold (CD133, *p* < 0.001), 1.4-fold (ALDHA1, *p* < 0.001), and 1.5-fold (CXCR4, *p* < 0.01) higher expression relative to 2D cultures. By day 7, expression further increased to 2.5-fold (CD24, *p* < 0.01), 3.1-fold (CD44, *p* < 0.001), 2.1-fold (CD133, *p* < 0.01), 2.1-fold (ALDHA1, *p* < 0.001), and 2.5-fold (CXCR4, *p* < 0.01).

In 3D-cultured HepG2 cells, at day 5, marker expression was elevated 1.2-fold (CD24), 1.7-fold (CD44, *p* < 0.05), 1.5-fold (CD133, *p* < 0.05), 1.9-fold (ALDHA1, *p* < 0.001), and 1.3-fold (CXCR4) compared with 2D cultures. At day 7, the increases were more evident: 1.7-fold (CD24, *p* < 0.01), 2.4-fold (CD44, *p* < 0.001), 1.9-fold (CD133, *p* < 0.01), 2.8-fold (ALDHA1, *p* < 0.001), and 1.8-fold (CXCR4, *p* < 0.05).

The expression of critical stemness- and pluripotency-regulating transcription factors, sex-determining region Y-box 2 (Sox2), octamer-binding transcription factor 4 (Oct4), homeobox Nanog transcription factor (Nanog), and Krüppel-like factor 4 (KLF4), was significantly enhanced in 3D cultures compared to 2D cultures across HLF, Huh7, and HepG2 cells (Figure 8B).

In HLF cells, Sox2, Nanog, Oct4, and KLF4 expression at day 5 increased 4.8-fold (*p* < 0.001), 1.7-fold (*p* < 0.01), 4.3-fold (*p* < 0.001), and 3.9-fold (*p* < 0.001), respectively, in 3D compared to 2D cultures. At day 7, expression further increased 6.4-fold (*p* < 0.001), 3.9-fold (*p* < 0.001), 5.8-fold (*p* < 0.001), and 5.5-fold (*p* < 0.01), respectively, in 3D cultures compared to 2D cultures.

In Huh7 cells, Sox2, Nanog, Oct4, and KLF4 expression increased 1.9-fold (*p* < 0.01), 1.7-fold (*p* < 0.001), 1.6-fold (*p* < 0.001), and 1.3-fold (*p* < 0.001), respectively, at day 5 in 3D compared to 2D cultures. At day 7, expression increased 3.6-fold (*p* < 0.01), 2.4-fold (*p* < 0.01), 2.9-fold (*p* < 0.001), and 2.0-fold (*p* < 0.01), respectively, in 3D cultures compared to 2D cultures.

For HepG2 cells, Sox2, Nanog, Oct4, and KLF4 expression increased 1.6-fold (*p* < 0.01), 2.0-fold (*p* < 0.05), 1.8-fold (*p* < 0.01), and 1.9-fold (*p* < 0.01) at day 5 in 3D compared to 2D cultures. At day 7, expression further increased 2.0-fold (*p* < 0.01), 2.5-fold (*p* < 0.05), 2.4-fold (*p* < 0.01), and 2.8-fold (*p* < 0.001), respectively, in 3D cultures compared to 2D cultures. Collectively, these findings demonstrate that 3D culture conditions markedly upregulate CSC marker expression in HCC cells, underscoring the tumor-like stemness phenotype promoted by the MCP-B hydrogel model.

### 2.6. Aggressiveness of HCC Cells Was Reinforced in MCP-B Hydrogels

Tumor cells cultured in 3D models, which more accurately replicate the in vivo tissue microenvironment, are believed to exhibit more aggressive phenotypes than those grown in traditional 2D monolayer cultures. We hypothesized that multicellular 3D HCC spheroids generated in MCP-B hydrogels would display accelerated progression compared to 2D-cultured cells. To explore the molecular mechanisms by which the 3D niche provided by MCP-B hydrogels influences malignant behavior and tumor progression in HCC, we evaluated the expression of key molecules associated with tumor aggressiveness.

**Figure 9:**
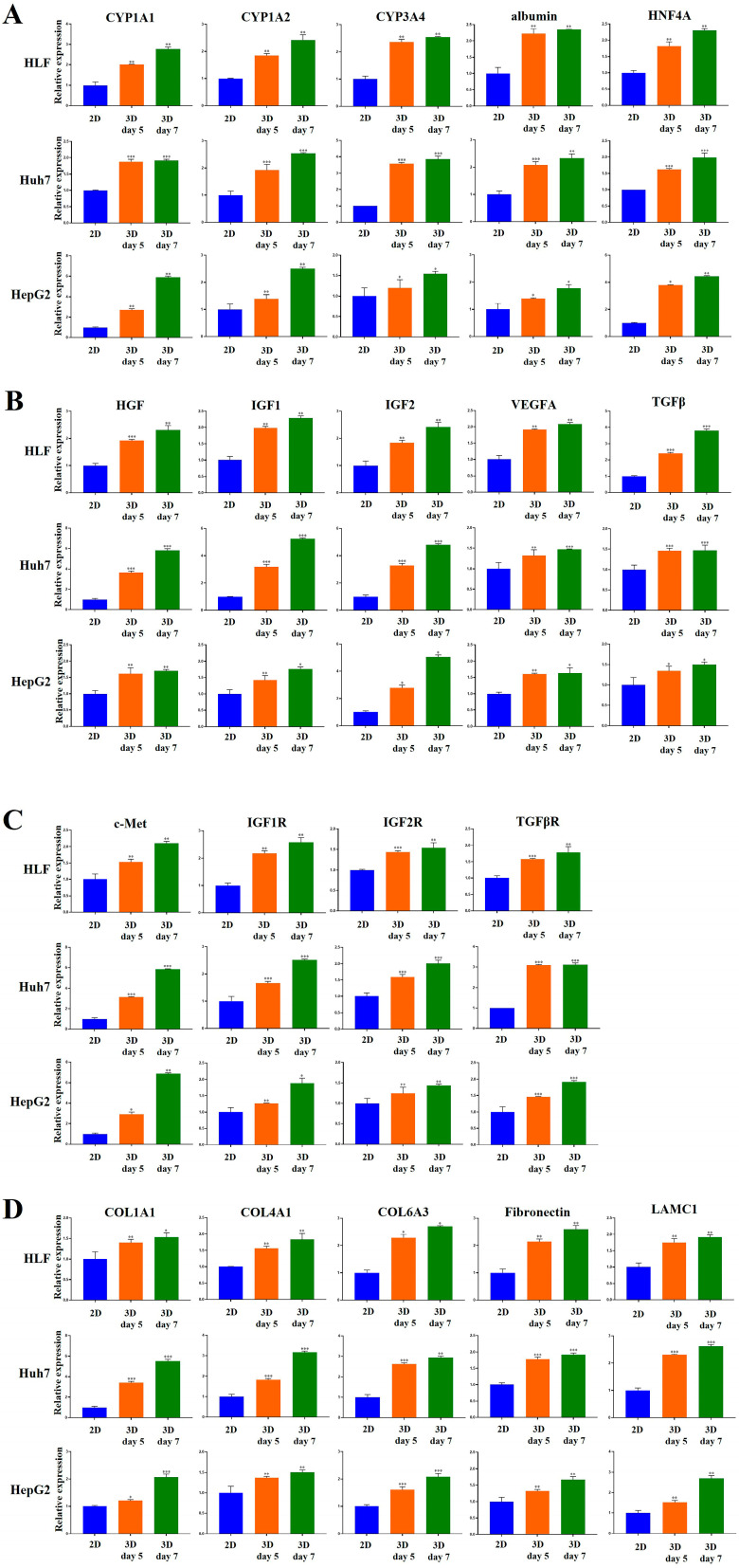
Gene expression analysis by quantitative reverse transcription PCR (qRT-PCR) of molecules associated with functional hepatocyte markers in HCC cell spheroids compared to 2D cell cultures. (**A**) Expression of genes associated with drug metabolism and CYP450 enzymes (CYP1A1, CYP1A2, CYP3A4) and liver-specific markers (albumin and HNF4A). (**B**) Expression of genes associated with growth factors (HGF, IGF1, IGF2, VEGFA, and TGFβ). (**C**) Expression of genes associated with growth factor receptors (c-Met, IGF1R, IGF2R, and TGFβR). (**D**) Expression of genes associated with ECM molecules (COL1A1, COL4A1, COL6A3, fibronectin, LAMC1). All measurements were performed in both 2D-cultured cells and 3D HCC spheroids. Bar graphs show mRNA levels normalized to GAPDH. Data are presented as the mean ± standard deviation of three independent experiments. * *p* < 0.05, ** *p* < 0.01, *** *p* < 0.001 vs. 2D.

First, we examined the gene expression of functional hepatocyte markers in HCC spheroids. In HLF cells, the gene expression of drug metabolism/CYP450 enzymes (CYP1A1, CYP1A2, CYP3A4) was significantly increased in 3D cultures compared to 2D cultures at both day 5 and day 7. At day 5, expression levels increased 2.0-fold (*p* < 0.01), 1.9-fold (*p* < 0.01), and 2.4-fold (*p* < 0.01), respectively (Figure 9A). At day 7, the fold increases were 2.8-fold (*p* < 0.01), 2.4-fold (*p* < 0.01), and 2.5-fold (*p* < 0.01), respectively (Figure 9A).

In Huh7 cells, CYP1A1, CYP1A2, and CYP3A4 expression was significantly higher in 3D than in 2D cultures at both day 5 and day 7. At day 5, expression increased 1.9-fold (*p* < 0.001), 1.9-fold (*p* < 0.001), and 3.6-fold (*p* < 0.001), respectively (Figure 9A). At day 7, expression further increased 1.9-fold (*p* < 0.001), 2.5-fold (*p* < 0.001), and 3.9-fold (*p* < 0.001), respectively (Figure 9A).

In HepG2 cells, CYP1A1, CYP1A2, and CYP3A4 expression in 3D cultures was significantly higher than that in 2D cultures at both day 5 and day 7. At day 5, expression increased 2.7-fold (*p* < 0.01), 1.4-fold (*p* < 0.01), and 1.2-fold (*p* < 0.05), respectively (Figure 9A). At day 7, expression further increased 5.9-fold (*p* < 0.01), 2.5-fold (*p* < 0.01), and 1.5-fold (*p* < 0.05), respectively (Figure 9A).

Furthermore, in HLF cells, the hepatic gene expression of albumin and HNF4A was increased 2.2-fold (*p* < 0.01) and 1.8-fold (*p* < 0.01), respectively, in 3D cultures compared to 2D cultures at day 5 (Figure 9A). At day 7, albumin and HNF4A gene expression was increased 2.4-fold (*p* < 0.01) and 2.3-fold (*p* < 0.01), respectively, in 3D cultures compared to 2D cultures (Figure 9A).

In Huh7 cells, albumin and HNF4A gene expression was increased 2.1-fold (*p* < 0.001) and 1.6-fold (*p* < 0.001), respectively, in 3D cultures compared to 2D cultures at day 5 (Figure 9A). At day 7, albumin and HNF4A gene expression increased 2.3-fold (*p* < 0.01) and 1.9-fold (*p* < 0.001), respectively, in 3D cultures compared to 2D cultures (Figure 9A).

In HepG2 cells, albumin and HNF4A gene expression was increased 1.4-fold (*p* < 0.05) and 3.8-fold (*p* < 0.05), respectively, in 3D cultures compared to 2D cultures at day 5 (Figure 9A). At day 7, albumin and HNF4A gene expression was increased 1.8-fold (*p* < 0.05) and 4.4-fold (*p* < 0.01), respectively, in 3D cultures compared to 2D cultures (Figure 9A).

These results suggest that 3D-cultured HCC cells in MCP-B hydrogels are functionally more efficient than 2D-cultured cells, highlighting their suitability for studies and modeling of HCC.

Next, we assessed the gene expression of growth factors and growth factor receptors associated with HCC development and malignancy in 3D HCC cell spheroids. In HLF cells, the gene expression of growth factors (HGF, IGF1, IGF2, VEGFA, and TGFβ) was increased 1.9-fold (*p* < 0.001), 1.9-fold (*p* < 0.01), 1.8-fold (*p* < 0.01), 1.9-fold (*p* < 0.01), and 2.4-fold (*p* < 0.001), respectively, in 3D cultures compared to 2D cultures at day 5 (Figure 9B). At day 7, the gene expression of these growth factors was increased 2.3-fold (*p* < 0.01), 2.3-fold (*p* < 0.01), 2.4-fold (*p* < 0.01), 2.1-fold (*p* < 0.01), and 3.8-fold (*p* < 0.001), respectively, in 3D cultures compared to 2D cultures (Figure 9B).

In Huh7 cells, the gene expression of HGF, IGF1, IGF2, VEGFA, and TGFβ was significantly increased in 3D cultures compared to 2D cultures at day 5. Specifically, expression increased 3.7-fold (*p* < 0.001), 3.2-fold (*p* < 0.001), 3.3-fold (*p* < 0.001), 1.3-fold (*p* < 0.01), and 1.5-fold (*p* < 0.001), respectively (Figure 9B). At day 7, HGF, IGF1, IGF2, VEGFA, and TGFβ expression levels were increased 5.8-fold (*p* < 0.001), 5.2-fold (*p* < 0.001), 4.8-fold (*p* < 0.001), 1.5-fold (*p* < 0.001), and 1.5-fold (*p* < 0.001), respectively, in 3D cultures compared to 2D cultures (Figure 9B).

In HepG2 cells, the gene expression of HGF, IGF1, IGF2, VEGFA, and TGFβ was significantly higher in 3D cultures compared to 2D cultures at day 5. Expression increased 1.6-fold (*p* < 0.01), 1.4-fold (*p* < 0.01), 2.8-fold (*p* < 0.05), 1.6-fold (*p* < 0.01), and 1.3-fold (*p* < 0.05), respectively (Figure 9B). At day 7, gene expression of these factors was increased 1.7-fold (*p* < 0.01), 1.8-fold (*p* < 0.05), 5.1-fold (*p* < 0.05), 1.6-fold (*p* < 0.05), and 1.5-fold (*p* < 0.05), respectively, in 3D cultures compared to 2D cultures (Figure 9B).

The gene expression of growth factor receptors (c-Met, IGF1R, IGF2R, and TGFβR) was significantly increased in 3D cultures compared to 2D cultures of HLF cells at both day 5 and day 7. At day 5, expression increased 1.5-fold (*p* < 0.01), 2.2-fold (*p* < 0.01), 1.4-fold (*p* < 0.001), and 1.6-fold (*p* < 0.001), respectively (Figure 9C). At day 7, fold increases were 2.1-fold (*p* < 0.01), 2.6-fold (*p* < 0.01), 1.5-fold (*p* < 0.01), and 1.8-fold (*p* < 0.01), respectively (Figure 9C).

In Huh7 cells, c-Met, IGF1R, IGF2R, and TGFβR gene expression was significantly increased in 3D cultures compared to 2D cultures at day 5 and day 7. At day 5, expression increased 3.1-fold (*p* < 0.001), 1.7-fold (*p* < 0.001), 1.6-fold (*p* < 0.001), and 3.1-fold (*p* < 0.001), respectively (Figure 9C). At day 7, expression further increased 5.8-fold (*p* < 0.001), 2.5-fold (*p* < 0.001), 2.0-fold (*p* < 0.001), and 3.1-fold (*p* < 0.001), respectively (Figure 9C).

In HepG2 cells, c-Met, IGF1R, IGF2R, and TGFβR gene expression was significantly higher in 3D cultures than in 2D cultures at both day 5 and day 7. At day 5, expression increased 2.9-fold (*p* < 0.05), 1.3-fold (*p* < 0.01), 1.2-fold (*p* < 0.01), and 1.5-fold (*p* < 0.001), respectively (Figure 9C). At day 7, gene expression was increased 6.9-fold (*p* < 0.01), 1.9-fold (*p* < 0.05), 1.4-fold (*p* < 0.01), and 1.9-fold (*p* < 0.001), respectively (Figure 9C).

In addition, the gene expression of liver ECM molecules, including COL1A1, COL4A1, COL6A3, fibronectin, and LAMC1, was examined in 3D-cultured HCC spheroids. In HLF cells, gene expression was increased at day 5 by 1.4-fold (*p* < 0.01), 1.6-fold (*p* < 0.01), 2.3-fold (*p* < 0.05), 2.1-fold (*p* < 0.01), and 1.8-fold (*p* < 0.01), respectively, compared to 2D cultures (Figure 9D). At day 7, expression further increased 1.5-fold (*p* < 0.05), 1.8-fold (*p* < 0.01), 2.7-fold (*p* < 0.05), 2.6-fold (*p* < 0.01), and 1.9-fold (*p* < 0.01), respectively (Figure 9D).

In Huh7 cells, COL1A1, COL4A1, COL6A3, fibronectin, and LAMC1 expression was significantly elevated at day 5, with fold increases of 3.4 (*p* < 0.001), 1.8 (*p* < 0.001), 2.6 (*p* < 0.001), 1.8 (*p* < 0.001), and 2.3 (*p* < 0.001), respectively, compared to 2D cultures (Figure 9D). At day 7, the increases were 5.5-fold (*p* < 0.001), 3.2-fold (*p* < 0.001), 2.9-fold (*p* < 0.01), 1.9-fold (*p* < 0.001), and 2.6-fold (*p* < 0.001), respectively (Figure 9D).

In HepG2 cells, COL1A1, COL4A1, COL6A3, fibronectin, and LAMC1 expression at day 5 increased 1.2-fold (*p* < 0.05), 1.4-fold (*p* < 0.01), 1.6-fold (*p* < 0.001), 1.3-fold (*p* < 0.01), and 1.5-fold (*p* < 0.01), respectively, compared to 2D cultures (Figure 9D). At day 7, expression increased 2.1-fold (*p* < 0.001), 1.5-fold (*p* < 0.01), 2.1-fold (*p* < 0.001), 1.7-fold (*p* < 0.01), and 2.7-fold (*p* < 0.01), respectively (Figure 9D).

Multiple lines of evidence indicate that epithelial–mesenchymal transition (EMT) is vital for tumor growth, progression, invasion, dissemination, metastasis, and drug resistance [48,49]. During metastasis, tumor cells degrade the ECM and invade blood vessels to establish secondary tumors, a process mediated largely by matrix metalloproteinases (MMPs) [50]. To further explore how the 3D microenvironment of MCP-B hydrogels contributes to tumor aggressiveness, we examined the expression of EMT-related molecules. Cells cultured in MCP-B hydrogels showed marked upregulation of Snail, Slug, Twist, Zeb1, Zeb2, vimentin, N-cadherin, and MMP9.

**Figure 10:**
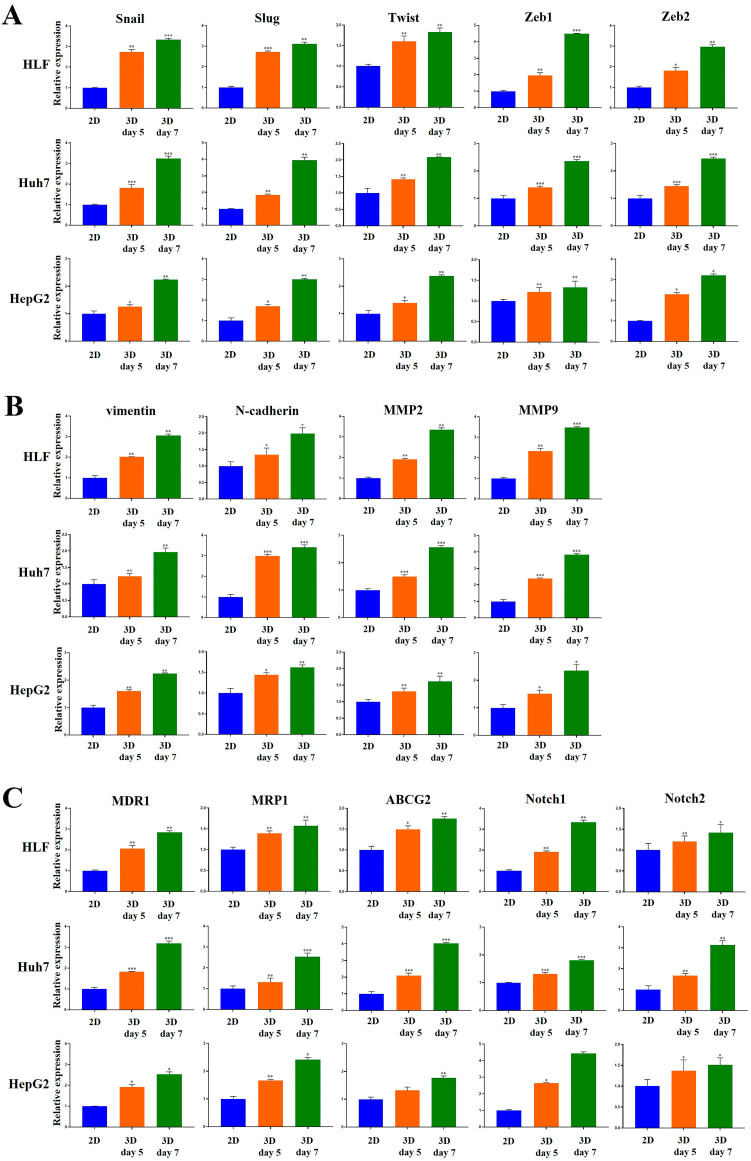
Gene expression analysis by quantitative reverse transcription PCR (qRT-PCR) of molecules associated with cancer aggressiveness in HCC cell spheroids compared with 2D cell cultures. (**A**) EMT-inducing transcription factors (Snail, Slug, Twist, Zeb1, and Zeb2). (**B**) EMT-associated molecules, including vimentin (major EMT biomarker), N-cadherin (mesenchymal marker), and MMP2/MMP9 (matrix metalloproteinases facilitating invasion and metastasis). (**C**) Genes linked to drug resistance (MDR1, MRP1, ABCG2, Notch1, Notch2). All measurements were performed in both 2D-cultured cells and 3D HCC spheroids. Bar graphs show mRNA levels normalized to GAPDH. Data are presented as the mean ± standard deviation of three independent experiments. * *p* < 0.05, ** *p* < 0.01, *** *p* < 0.001 vs. 2D.

The gene expression of Snail, Slug, Twist, Zeb1, and Zeb2 significantly increased in 3D cultures compared to 2D cultures for HLF cells on day 5. Specifically, expression increased 2.7-fold (*p* < 0.01), 2.7-fold (*p* < 0.001), 1.6-fold (*p* < 0.01), 1.9-fold (*p* < 0.01), and 1.8-fold (*p* < 0.05), respectively (Figure 10A). At day 7, these increases were 3.3-fold (*p* < 0.001), 3.1-fold (*p* < 0.01), 1.8-fold (*p* < 0.01), 4.5-fold (*p* < 0.001), and 2.9-fold (*p* < 0.01), respectively (Figure 10A).

In Huh7 cells, Snail, Slug, Twist, Zeb1, and Zeb2 gene expression was also increased at day 5, with increases of 1.8-fold (*p* < 0.001), 1.8-fold (*p* < 0.01), 1.4-fold (*p* < 0.01), 1.4-fold (*p* < 0.001), and 1.4-fold (*p* < 0.001), respectively (Figure 10A). At day 7, expression increased 3.2-fold (*p* < 0.001), 3.9-fold (*p* < 0.01), 2.1-fold (*p* < 0.01), 2.4-fold (*p* < 0.001), and 2.5-fold (*p* < 0.001), respectively (Figure 10A).

In HepG2 cells, the gene expression of Snail, Slug, Twist, Zeb1, and Zeb2 was increased at day 5, with fold increases of 1.3 (*p* < 0.05), 1.7 (*p* < 0.05), 1.4 (*p* < 0.05), 1.2 (*p* < 0.01), and 2.3 (*p* < 0.05), respectively (Figure 10A). At day 7, expression was increased 2.2-fold (*p* < 0.01), 3.0-fold (*p* < 0.01), 2.4-fold (*p* < 0.01), 1.3-fold (*p* < 0.01), and 3.2-fold (*p* < 0.05), respectively (Figure 10A).

The gene expression of vimentin, N-cadherin, MMP2, and MMP9 was significantly increased in HLF cells in 3D cultures compared to 2D cultures at both day 5 and day 7. At day 5, expression increased 2.0-fold (*p* < 0.01), 1.4-fold (*p* < 0.05), 1.9-fold (*p* < 0.01), and 2.3-fold (*p* < 0.01), respectively (Figure 10B). At day 7, the increases were 3.1-fold (*p* < 0.01), 1.9-fold (*p* < 0.05), 3.3-fold (*p* < 0.01), and 3.5-fold (*p* < 0.001), respectively (Figure 10B).

In Huh7 cells, vimentin, N-cadherin, MMP2, and MMP9 gene expression at day 5 increased 1.2-fold (*p* < 0.01), 3.0-fold (*p* < 0.001), 1.5-fold (*p* < 0.001), and 2.4-fold (*p* < 0.001), respectively (Figure 10B). At day 7, the fold increases were 1.9-fold (*p* < 0.01), 3.4-fold (*p* < 0.001), 2.6-fold (*p* < 0.001), and 3.8-fold (*p* < 0.001), respectively (Figure 10B).

In HepG2 cells, vimentin, N-cadherin, MMP2, and MMP9 gene expression at day 5 increased 1.6-fold (*p* < 0.01), 1.4-fold (*p* < 0.05), 1.3-fold (*p* < 0.01), and 1.5-fold (*p* < 0.05), respectively (Figure 10B). At day 7, expression increased 2.2-fold (*p* < 0.01), 1.6-fold (*p* < 0.01), 1.6-fold (*p* < 0.01), and 2.3-fold (*p* < 0.05), respectively (Figure 10B).

To investigate the effects of the 3D niche provided by MCP-B hydrogels on multidrug-resistance-related genes, key drivers of chemoresistance in cancer, we compared their expression in cells grown in 2D vs. 3D cultures.

In HLF cells, MDR1 and MRP1 expression was upregulated by 2.1-fold (*p* < 0.01) and 1.4-fold (*p* < 0.01), respectively, at day 5 in 3D cultures compared to 2D controls (Figure 10C). By day 7, MDR1 and MRP1 expression further increased to 2.9-fold (*p* < 0.01) and 1.6-fold (*p* < 0.01), respectively (Figure 10C).

In Huh7 cells, MDR1 and MRP1 were upregulated by 1.8-fold (*p* < 0.001) and 1.3-fold (*p* < 0.01), respectively, at day 5 in 3D cultures. At day 7, their expression rose further, reaching 3.2-fold (*p* < 0.001) and 2.5-fold (*p* < 0.001), respectively (Figure 10C).

In HepG2 cells, MDR1 and MRP1 expression was upregulated by 1.9-fold (*p* < 0.05) and 1.7-fold (*p* < 0.01), respectively, in 3D cultures at day 5. By day 7, expression increased to 2.5-fold (*p* < 0.05) and 2.4-fold (*p* < 0.05), respectively (Figure 10C).

We also examined ABCG2, a key regulator of stemness and pluripotency in HCC. In HLF cells, ABCG2 expression was elevated by 1.5-fold (*p* < 0.05) and 1.8-fold (*p* < 0.01) in 3D cultures compared with 2D-cultured controls at days 5 and 7, respectively. In Huh7 cells, ABCG2 expression was upregulated, showing 2.1-fold (*p* < 0.001) and 4.0-fold (*p* < 0.001) increases at days 5 and 7, respectively. In HepG2 cells, ABCG2 was upregulated by 1.3-fold and 1.8-fold (*p* < 0.01) in 3D cultures at days 5 and 7, respectively (Figure 10C).

To further explore how the 3D microenvironment in MCP-B hydrogels regulates tumor aggressiveness in HCC, we evaluated the expression of Notch1 and Notch2. In HLF cells, Notch1 expression was upregulated by 1.9-fold (*p* < 0.01) and 3.3-fold (*p* < 0.01) in 3D cultures compared to 2D cultures at days 5 and 7, respectively (Figure 10C). In Huh7 cells, Notch1 expression was also increased by 1.3-fold (*p* < 0.001) and 1.8-fold (*p* < 0.001) at days 5 and 7, respectively, in 3D cultures compared to 2D controls (Figure 10C). In HepG2 cells, Notch1 expression was upregulated by 2.6-fold (*p* < 0.05) and 4.4-fold at days 5 and 7, respectively, in 3D cultures compared to 2D cultures (Figure 10C). For Notch2, HLF cells showed upregulation by 1.2-fold (*p* < 0.01) and 1.4-fold (*p* < 0.05) in 3D cultures compared to 2D cultures at days 5 and 7, respectively (Figure 10C). Notch2 gene expression in Huh7 cells was also increased by 1.7-fold (*p* < 0.01) and 3.1-fold (*p* < 0.01) in 3D cultures compared to 2D cultures at days 5 and 7, respectively (Figure 10C). In HepG2 cells, Notch2 expression was upregulated by 1.4-fold (*p* < 0.05) and 1.5-fold (*p* < 0.05) in 3D cultures compared to 2D cultures at days 5 and 7, respectively (Figure 10C).

**Figure 11:**
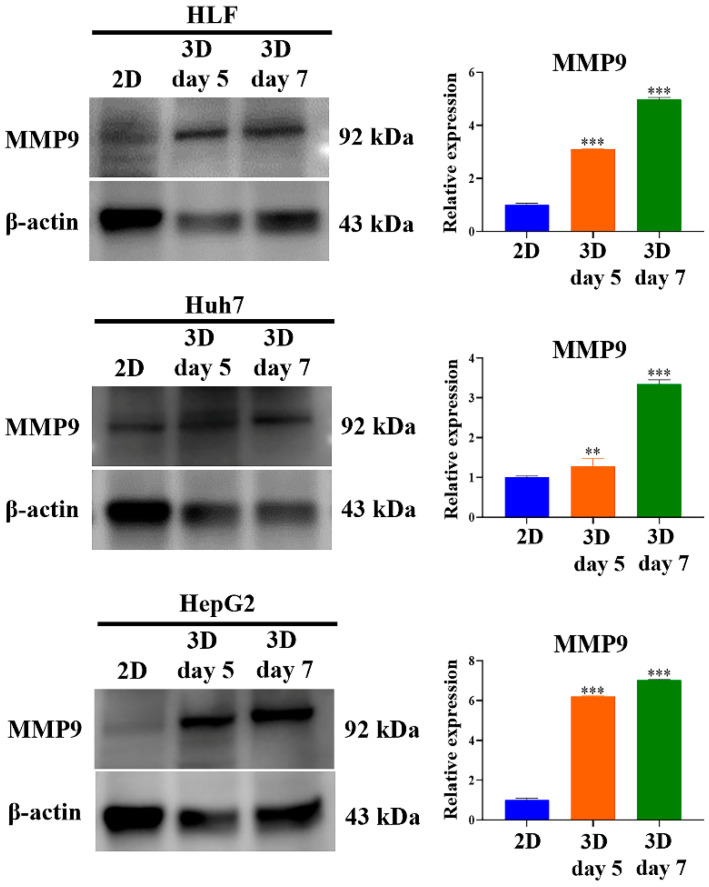
Western blot analysis of MMP9 in HLF, Huh7, and HepG2 cells cultured under 2D and 3D conditions. Bar graphs represent densitometric quantification of protein levels normalized to β-actin. Data are shown as mean ± SD. ** *p* < 0.01, *** *p* < 0.001 vs. 2D.

Western blot analysis showed that HCC cells cultured as 3D spheroids in MCP-B hydrogels exhibited higher levels of MMP9 compared with 2D-cultured cells. In HLF cells, 3D culture induced a 3.1-fold and 4.9-fold increase at days 5 and 7, respectively (*p* < 0.001 each). In Huh7 cells, MMP9 levels were increased 1.3-fold (*p* < 0.01) and 3.3-fold (*p* < 0.001) at days 5 and 7, respectively. In HepG2 cells, 3D culture induced 6.2-fold (*p* < 0.001) and 7.0-fold (*p* < 0.001) increases at days 5 and 7, respectively, compared with 2D controls (Figure 11).

### 2.7. MCP-B Hydrogels Reduced Human HCC Cell Apoptosis

**Figure 12:**
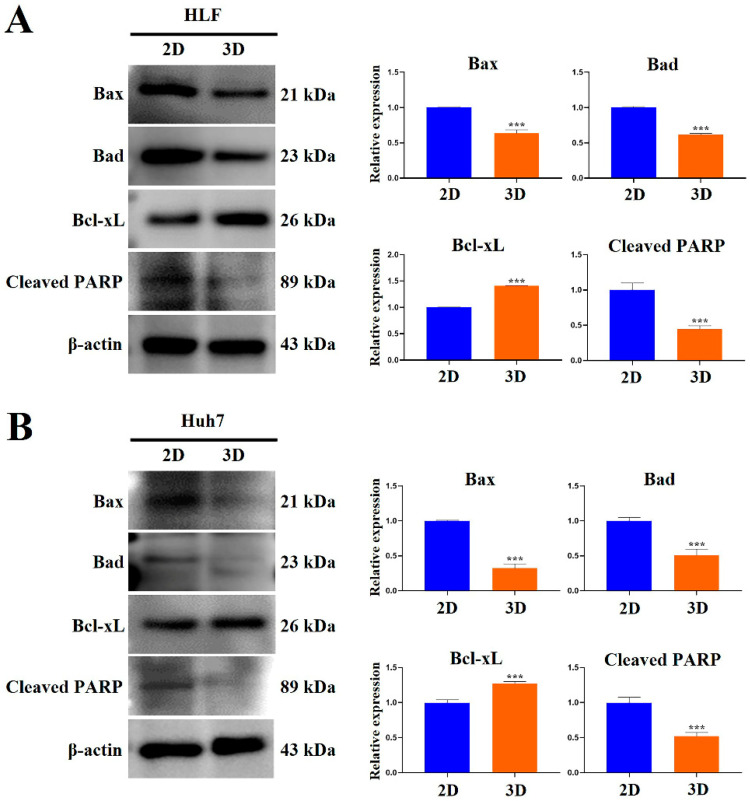
Apoptosis in HCC cell spheroids compared with 2D cell cultures. Western blot analysis of apoptotic proteins in HLF (**A**) and Huh7 (**B**) cells grown in 2D or 3D. Bar graphs show densitometry quantification of protein levels normalized to β-actin. Data are presented as mean ± SD from three independent experiments. *** *p* < 0.001 vs. 2D.

Western blot analysis showed that HCC cells cultured as 3D spheroids exhibited reduced levels of apoptosis compared with 2D-cultured cells (Figure 11). In HLF cells, 3D culture significantly reduced the levels of the pro-apoptotic proteins Bax and Bad by 0.6-fold (*p* < 0.001 each) compared to 2D controls, while the anti-apoptotic protein Bcl-xL was increased 1.4-fold (*p* < 0.001) (Figure 12A). Consistently, Cleaved PARP levels were markedly suppressed by 0.4-fold (*p* < 0.001) in 3D-cultured HLF cells relative to 2D controls (Figure 12A).

In Huh7 cells, Bax and Bad were significantly reduced by 0.3-fold (*p* < 0.001) and 0.5-fold (*p* < 0.001), respectively, in 3D cultures compared to 2D controls. In contrast, Bcl-xL expression was increased 1.3-fold (*p* < 0.001) in 3D cultured Huh7 cells. Similarly, Cleaved PARP was suppressed by 0.5-fold (*p* < 0.001) in 3D cultures compared with 2D (Figure 12B). Together, these results indicate that MCP-B 3D hydrogels promote an anti-apoptotic phenotype in human HCC cells.

### 2.8. HCC Stem Cell Biomarker Expression Was Augmented in MCP-B Hydrogels

In addition to gene expression analyses of cancer stemness-associated markers, we evaluated the expression of CSC biomarkers to determine the CSC enrichment efficiency of our 3D HCC cell culture model. Flow cytometry was used to assess CSC biomarker expression in HCC cells cultured under 2D and 3D conditions. Typical HCC-related CSC surface markers, such as ALDHA1, CD44, CD90, CD117, EpCAM, and cytokeratin 19 (CK19), were evaluated in human HCC cells (HLF, HepG2, Huh7, and PLC/PRF/5).

**Figure 13:**
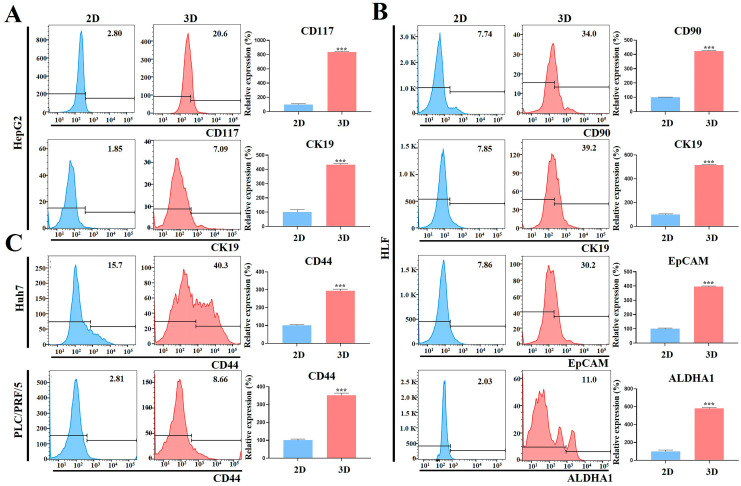
Cancer stem cell surface biomarker expression in HCC cells cultured in standard plastic plates (2D) or MCP-B hydrogels (3D). (**A**) Flow cytometry of HepG2 cells on day 7 showing significantly higher CD117 and CK19 expression in 3D spheroids compared to 2D cultures. (**B**) HLF cells on day 7 showing significantly higher CD90, CK19, EpCAM, and ALDHA1 expression in 3D spheroids compared to 2D cultures. (**C**) Huh7 and PLC/PRF/5 cells on day 7 showing significantly higher CD44 expression in 3D spheroids compared to 2D cultures. Data represent the mean ± standard deviation of three independent experiments. *** *p* < 0.001 vs. 2D.

In HepG2, flow cytometric analysis on day 7 showed that the percentages of CD117- and CK19-positive cells were higher in 3D spheroids than in 2D cultures, by 8.4-fold (*p* < 0.001) and 4.3-fold (*p* < 0.001), respectively (Figure 13A). In HLF cells, the percentages of CD90-, CK19-, EpCAM-, and ALDHA1-positive cells were higher in 3D spheroids than in 2D cultures, by 4.2-fold (*p* < 0.001), 5.2-fold (*p* < 0.001), 4.0-fold (*p* < 0.001), and 5.8-fold (*p* < 0.001), respectively (Figure 13B). In Huh7 and PLC/PRF/5 cells, the percentages of CD44-positive cells were higher in 3D spheroids than in 2D cultures, by 2.9-fold (*p* < 0.001) and 3.5-fold (*p* < 0.001), respectively (Figure 13C).

## 3. Discussion

Few malignancies embody such relentless progression as LC, whose growing global burden demands breakthroughs in both scientific understanding and therapeutic intervention [51,52]. Here, we show that 3D spheroid cultures of HCC cells within biomimetic MCP-B hydrogels recapitulate a broad spectrum of tumor aggressiveness, combining invasive growth, survival advantages, and sustained activation of EMT-, stemness-, and drug-resistance programs, to a degree far surpassing that of conventional 2D systems. These findings challenge the adequacy of traditional monolayer models and underscore the necessity of physiologically relevant 3D platforms as tools for unraveling tumor complexity and accelerating therapeutic discovery. Our culture system not only elucidates fundamental mechanisms of HCC malignancy but also opens new avenues for therapeutic targeting, establishing a benchmark for 3D in vitro HCC modeling.

Effective treatment strategies for HCC remain limited, largely because of the persistent proliferation of tumor cells and the complexity of its pathogenesis, which unfolds through a multistep biological process during tumor development and progression [53,54,55,56,57]. Deciphering the cellular and molecular landscape of HCC progression remains a major challenge, underscoring the need for models that faithfully capture the phenotypic and molecular characteristics of the disease [58].

Most current in vitro studies rely on 2D cultures, which are cost-effective and straightforward but fail to replicate the complex tissue architecture, cell–cell and cell–matrix interactions, and biochemical signaling of native tumor tissue [59]. In contrast, 3D culture systems more accurately mimic essential in vivo biological processes, including gene and protein expression, cell survival, proliferation, adhesion, migration, development, and differentiation, both functionally and morphologically [60,61]. Consequently, 3D platforms have emerged as powerful tools for constructing tumor models that closely recapitulate in vivo cancer behavior and provide a multidimensional perspective on tumor biology and therapeutic responses [62,63,64]. Transitioning from 2D to 3D systems is therefore essential for improving translational relevance in HCC research [65,66].

Three-dimensional culture systems provide advantages over conventional 2D models. Scaffold-based approaches strengthen cell–cell and cell–matrix interactions, yielding more physiologically relevant drug responses and gene expression profiles [26,27,28,29,30,31,32,33,67,68,69]. Among biomaterials, Matrigel—derived from Engelbreth–Holm–Swarm mouse sarcoma secretions—has enabled the generation of organoid models for multiple tissues and organs [70,71,72,73,74,75,76,77,78]. However, its complexity (>1800 proteins) and undefined composition complicate identification of the specific cues regulating spheroid and organoid development [79]. In contrast, collagen, a principal ECM component, is widely applied as a scaffold because it provides a more defined and controllable environment for 3D culture and tissue engineering [80]. Compared with these systems, our 3D HCC culture model using MCP-B hydrogels offers distinct advantages: it is robust, biomimetic, and can be applied for efficient, rapid, and cost-effective ex vivo drug sensitivity testing as well as mechanistic studies. Thus, the MCP-B hydrogel system represents a promising platform for studying cancer biology and evaluating therapeutic strategies.

Compared with 2D monolayers, our 3D spheroids exhibited markedly enhanced tumor-like behaviors, including increased proliferation, clonogenicity, migration, invasion, and chemoresistance, along with molecular signatures characteristic of aggressive disease. These included elevated expression of multidrug resistance and cancer stemness-associated genes (MDR1, MRP1, ABCG2, CD24, CD44, CD133, ALDHA1, CXCR4, Sox2, Oct4, Nanog, and KLF4), upregulated anti-apoptotic molecules (Bcl-xL) with concomitant downregulation of pro-apoptotic markers (Bax, Bad, and Cleaved PARP), and retention of hepatocyte functional markers (albumin, CYP1A1, CYP1A2, CYP3A4, and HNF4A). The spheroids also displayed heightened EMT activity, activation of growth factor signaling, and expression of tumor progression markers (Snail, Slug, Twist, Zeb1, Zeb2, vimentin, N-cadherin, MMP2, MMP9, HGF, IGF1, IGF2, VEGFA, TGFβ, c-Met, IGF1R, IGF2R, TGFβR, Notch1, and Notch2), together with increased ECM and fibrosis-related molecules (COL1A1, COL4A1, COL6A3, fibronectin, and LAMC1).

The heightened expression of key hepatocyte functional markers in 3D-cultured cells compared to 2D-cultured cells suggests that our 3D HCC model more closely mimics the in vivo HCC environment, providing a physiologically relevant platform that may yield more predictive results for studying HCC biology and evaluating therapeutic agents. These findings are consistent with reports by [81,82,83] who demonstrated that HCC spheroids exhibit higher mRNA levels of hepatocyte functional markers such as CYP1A1, CYP1A2, and CYP3A4 compared with 2D cultures [84,85,86,87,88]. Additionally, Kato et al. (2014) reported that albumin, HNF4A, and CYP enzyme expression levels were higher in 3D cultures than in conventional 2D conditions [89].

Cell proliferation is a fundamental hallmark of cancer, driving both tumor initiation and progression. In normal tissues, growth is tightly regulated by intrinsic and extrinsic signals, but cancer cells evade these controls and undergo unchecked, continuous division. This uncontrolled expansion increases tumor burden, contributes to adverse effects on the host, and fuels metastatic spread. Malignant cells therefore acquire traits that allow survival beyond their normal lifespan and enable persistent proliferation. Accordingly, most current cancer therapies, particularly cytotoxic drugs, aim to suppress proliferation by killing rapidly dividing cells and preventing further accumulation.

Despite this therapeutic focus, conventional 2D culture systems provide only a limited model of cancer cell proliferation. In 2D culture, cells expand on a flat surface until they form a confluent monolayer, where uniform access to nutrients and growth factors promotes rapid proliferation but supports only short-term growth. Once confluence is reached, growth is restricted by the limited surface area of the dish, and contact inhibition halts further division, limiting its ability to model sustained tumor expansion [90]. By contrast, 3D culture systems allow cells to form spheroids, supporting continuous growth over longer periods and better mimicking the dynamic tumor microenvironment [91,92].

In our study, HCC cell spheroids generated within MCP-B hydrogels exhibited enhanced proliferation, increased cell cycle activity, and greater colony-forming capacity compared with 2D cultures. These findings indicate that our hydrogel-based 3D system provides a favorable environment for sustained HCC cell growth, offering a more physiologically relevant platform for investigating tumor biology and evaluating proliferation-targeted therapies.

Multidrug resistance (MDR) remains a major barrier to effective HCC treatment [93]. Among the most common mechanisms is drug efflux mediated by adenosine triphosphate (ATP)-binding cassette (ABC) transporter proteins such as MDR1 and MRP1, which use ATP hydrolysis to actively remove cytotoxic agents from cells and lower intracellular drug concentrations [94,95,96]. Additional resistance mechanisms include impaired apoptosis and dysregulated cell cycle control [97]. In line with these concepts, we observed pronounced chemoresistance in HCC cells grown in MCP-B hydrogels, accompanied by upregulation of MDR1 and MRP1, highlighting the model’s capacity to reflect clinically relevant drug resistance phenotypes more faithfully than 2D cultures.

Spheroid size in our cultures exceeded 190 µm by day 12, a threshold at which diffusion limitations generate hypoxic zones and necrotic cores [94,98,99]. Hypoxia is a key driver of therapeutic resistance, partly through activation of signaling pathways that enhance CSC maintenance and resistance to chemotherapy and radiotherapy [98]. Thus, the MCP-B hydrogel system not only supports physiologically relevant spheroid growth but also recapitulates microenvironmental stressors with direct clinical implications.

A notable feature of our model is its efficiency in enriching liver cancer stem cells (LCSCs). CSCs possess self-renewal capacity, multipotency, and resistance to conventional therapies, making them central to tumor initiation, recurrence, and metastasis [100,101,102]. LCSCs are defined by transcription factors such as Oct4, Sox2, Nanog, and SALL4 [103], as well as surface markers including CD24, CD44, CD133, ALDHA1, and CXCR4. In our system, CSC enrichment was evidenced by increased expression of these markers and associated genes, including MDR1, MRP1, and ABCG2. Notably, ABCG2 is regulated by Oct4 [104], ABCB5 by granulin–epithelin precursor [105], and MDR1 by c-Myc, Nanog, or IL-8 [106,107,108], all of which enhance drug efflux. Literature further supports functional roles for individual markers: CD24 overexpression correlates with chemoresistance and poor prognosis [109,110]; CD44 promotes HCC progression via YAP signaling [111]; CD133 identifies chemoresistant subpopulations [112,113,114,115,116]; and ALDH1 is linked to tumorigenicity and adverse prognosis [117,118,119,120,121,122,123,124,125]. Together, these findings confirm that MCP-B hydrogels offer a reliable platform for CSC induction and enrichment, enabling mechanistic studies and preclinical evaluation of CSC-targeted therapies.

EMT emerged as another prominent feature in our model. EMT describes the transition from an epithelial to a mesenchymal phenotype, characterized by loss of cell–cell adhesion, polarity, and epithelial markers (E-cadherin, β-catenin) along with the gain of mesenchymal traits (N-cadherin, vimentin) [126,127,128]. This process is regulated by transcription factors such as Twist, Snail, Slug, Zeb1, and Zeb2 [129], all of which were elevated in our 3D spheroids. EMT contributes to invasion, metastasis, apoptosis resistance, and ECM production [48,49,127], and is mechanistically linked to CSC generation. In addition, the Notch signaling pathway, also upregulated in our model, promotes proliferation, aggressiveness, stem cell maintenance, and chemoresistance by inducing EMT, generating CSCs, and increasing MDR protein expression [130,131,132,133,134]. Moreover, matrix metalloproteinases (MMP2, MMP9)—key mediators of ECM remodeling and metastasis—were significantly elevated, reinforcing the model’s relevance for studying EMT-driven tumor progression [135].

Thus, our findings reveal that HCC spheroids cultured within MCP-B hydrogels acquire markedly enhanced migratory and invasive behavior compared with 2D cultures, underscoring the hydrogel’s capacity to recapitulate the aggressive phenotypes that drive tumor progression and metastasis in patients.

The extracellular matrix (ECM) remodeling also emerged as a critical hallmark in our model. The ECM is not a passive scaffold but a dynamic 3D network that provides structural support as well as biochemical and mechanical cues regulating cell behavior and tissue homeostasis. In HCC, ECM composition and stiffness are profoundly altered, driving tumor cell proliferation, invasion, and resistance to therapy [136,137].

Our spheroids exhibited upregulation of major ECM components, including COL1A1, COL4A1, COL6A3, fibronectin, and LAMC1, reflecting a fibrotic and pro-tumorigenic microenvironment. Among these, type I collagen (COL1A1) is especially critical, as it promotes EMT, tumorigenesis, metastasis, and resistance to apoptosis and chemotherapy across multiple cancer types [138]. Elevated COL1A1 levels in HCC are associated with poor prognosis, making it both a biomarker and a potential therapeutic target [139]. Mechanistically, collagen I enhances stemness (Sox2, Oct4, CD133) through Slug-dependent EMT and, via DDR1–CD44 signaling, disrupts Hippo pathway regulation to activate YAP, thereby reinforcing therapeutic resistance and tumor progression [140]. Notably, silencing COL1A1 by siRNA has been shown to impair HCC cell proliferation, stemness, motility, and tumorsphere formation [139].

Fibronectin, another key glycoprotein, rapidly accumulates upon liver injury and chronic inflammation, serving as a biomarker for early-stage HCC and chronic liver disease [141]. Through interactions with integrins and collagens, fibronectin promotes tumor angiogenesis, cell adhesion, and proliferation [141]. LAMC1, also markedly upregulated in HCC tumors, has been associated with enhanced metastasis and poor patient outcomes [142].

Beyond these structural roles, the ECM actively shapes the tumor microenvironment by regulating immune cell infiltration, vascular remodeling, and drug penetration. Collectively, these findings highlight that the MCP-B hydrogel platform faithfully recapitulates ECM deposition and remodeling, enabling investigation of ECM–tumor crosstalk and providing a versatile system for evaluating ECM-targeted therapeutic interventions.

Our study demonstrates important advancements but also has a limitation that should be acknowledged. While we observed significant mRNA expression changes that highlight the advantages of 3D cell culture with MCP-B hydrogels over 2D monolayer culture in recapitulating key features of tumor biology, these findings have not yet been validated at the protein level or in terms of functional activity. Future studies will be needed to confirm the observed mRNA changes through protein-level and functional analyses, thereby providing a more comprehensive understanding of the physiological relevance of MCP-B hydrogels for 3D HCC culture.

In summary, the MCP-B hydrogel-based 3D HCC culture system outperforms 2D cultures by recapitulating cellular functionality and reproducing multiple hallmarks of HCC malignancy, including CSC enrichment, EMT induction, ECM remodeling, hypoxia formation, and multidrug resistance. This physiologically relevant platform supports mechanistic investigations into tumor progression, facilitates drug screening, and provides a valuable preclinical model for developing targeted therapies, particularly those aimed at CSCs and EMT-associated pathways. Future studies integrating this system with co-culture approaches, patient-derived cells, or immune components could further enhance its translational potential and contribute to the development of more effective strategies against HCC.

## 4. Materials and Methods

### 4.1. Cell Culture

Human HCC cell lines HLF, Huh7, HepG2, PLC/PRF/5, SNU449, and SNU475 were obtained from the American Type Culture Collection (Manassas, VA, USA). HLF, Huh7, and HepG2 cells were cultured in Dulbecco’s Modified Eagle Medium (DMEM; Hyclone, Chicago, IL, USA), whereas PLC/PRF/5, SNU449, and SNU475 were maintained in Roswell Park Memorial Institute (RPMI) 1640 medium (Hyclone). All media were supplemented with 10% fetal bovine serum (FBS; Welgene, Daegu, Republic of Korea) and 1% penicillin–streptomycin (Thermo Fisher Scientific, Waltham, MA, USA). Cells were incubated at 37 °C in a humidified atmosphere with 5% CO_2_. Subconfluent cells were passaged using trypsin-EDTA (Welgene) and harvested for experiments. The medium was refreshed every 2–3 days.

### 4.2. Synthesis of Hydrogels for 3D Cell Culture

MCPs are known for their excellent biocompatibility and ability to form hydrogels under mild conditions, making them suitable for 3D cell culture applications. The MCPs used in this study were derived from Alaskan cod (Natural Force, Jacksonville, FL, USA) and consist primarily of type I collagen peptides with a molecular weight range of 500–3000 Da. They are highly soluble in water and rich in glycine, proline, and hydroxyproline, key amino acids that contribute to collagen’s structural stability and bioactivity. MCP-B hydrogels for 3D cell culture were prepared as previously described [143,144]. Briefly, agarose (Affymetrix, Cleveland, OH, USA) was dissolved in distilled water and heated to 100 °C to obtain a 2% agarose stock solution. The peptides were dissolved in distilled water at 0.3 g/mL with vortexing to prepare a 30% (*w*/*v*) stock solution. This stock solution was stored at 4 °C to maintain peptide integrity. For hydrogel preparation, 592 μL of cell suspension (1 × 10^5^ cells/mL in culture medium) was mixed with 333 μL of the 30% MCP stock solution at room temperature, yielding a 10% MCP solution containing cells. This mixture was then gently combined with 75 μL of 2% agarose solution maintained at 35–40 °C to avoid thermal damage to the cells.

The resulting hydrogel–cell suspensions were briefly vortexed, transferred into 1 mL syringes, and incubated at 4 °C for 5–10 min to allow gelation. The solidified hydrogels were then placed into 24-well plates (SPL Life Sciences, Pocheon, Republic of Korea) containing 1.5 mL of either RPMI 1640 or DMEM (both from HyClone). Cultures were maintained at 37 °C in a humidified 5% CO_2_ incubator, and the medium was replaced every 2 days.

### 4.3. Spheroid Growth Assay

To assess the effects of MCP-B hydrogels on multicellular spheroid formation and growth, HLF, Huh7, and HepG2 cells were cultured for 1, 3, 5, 7, 10, and 12 days. At each time point, spheroid size was evaluated using a phase-contrast microscope (EVOS M7000, Thermo Fisher Scientific). For each condition, at least 20 spheroids per hydrogel were imaged, and their diameters were quantified. The spheroid diameter was defined as the mean of two diameters measured at 90° angular intervals across the spheroid outline, passing through the centroid, to ensure accurate and reproducible size determination.

### 4.4. Cell Proliferation Assay

Cell proliferation in 2D and 3D cultures was evaluated as previously described [144], with minor modifications. For 2D culture, HLF, Huh7, and HepG2 cells were seeded at a density of approximately 1 × 10^4^ cells/well in 96-well plates (SPL Life Sciences). For 3D culture, cells were encapsulated in MCP-B hydrogels at a density of 1 × 10^5^ cells/mL. Both culture systems were maintained in complete medium supplemented with 10% FBS (Welgene) for 1, 3, 5, 7, 10, and 12 days.

Cell proliferation was assessed using the WST-1 colorimetric assay (Daeil Lab Service, Seoul, Republic of Korea), following the manufacturer’s protocol. Briefly, culture wells were washed with phosphate-buffered saline (PBS), followed by the addition of 10 μL of WST-1 reagent per well. Plates were incubated for 1 h at 37 °C in a humidified 5% CO_2_ atmosphere, after which the absorbance of formazan was measured at 450 nm using a microplate reader (Tecan, Männedorf, Switzerland).

Cell viability was expressed as a percentage relative to the 2D control population. In parallel, cell morphology and spheroid size were monitored at the indicated time points using a phase-contrast microscope (EVOS M7000, Thermo Fisher Scientific). All experiments were performed independently in triplicate to ensure reproducibility.

### 4.5. Colony-Forming Assay

The colony-forming ability of HLF and Huh7 cells was evaluated following 2D and 3D culture. Cells harvested after 7 days of culture were seeded into 6-well plates (SPL Life Sciences) at a density of 400 cells/well, as described previously [144]. The cells were maintained for an additional 10 and 14 days, during which visible colonies developed.

At the end point, colonies were fixed with 100% methanol for 20 min at −20 °C, washed with PBS, and stained with 0.5% crystal violet solution (Sigma-Aldrich, St. Louis, MO, USA) for 10 min. Following three PBS washes, plates were air-dried at room temperature. Colonies were counted using a phase-contrast microscope (EVOS M7000, Thermo Fisher Scientific) to quantify colony-forming units. All experiments were performed independently in triplicate to ensure reproducibility.

### 4.6. Wound-Healing Assay

The migratory capacity of HLF and Huh7 cells was evaluated using a wound-healing assay. Cells previously cultured in MCP-B hydrogels for 7 days were harvested and seeded into 6-well plates (SPL Life Sciences) at a density of 1 × 10^6^ cells/well. Cells maintained under conventional 2D culture served as controls. Once both 2D- and 3D-derived cells reached confluence, the medium was replaced with starvation medium containing 2% FBS (Welgene) to minimize proliferation-driven wound closure. A uniform scratch was then made across the monolayer using a cell scratcher (SPL Life Sciences). Detached cells and debris were removed by gentle PBS washing, leaving a clear wound gap. Wound closure was monitored at defined time points, and images were captured using a phase-contrast microscope (EVOS M7000, Thermo Fisher Scientific). The assay was performed independently in triplicate to ensure reproducibility.

### 4.7. Hydrogel Invasion Assay

The invasive capacity of HCC cells was assessed using a hydrogel-based invasion assay in 96-well plates (SPL Life Sciences). Briefly, 1 × 10^4^ cells/well were harvested, resuspended in RPMI 1640 (Hyclone) or DMEM (Hyclone), and seeded onto the upper surface of MCP-B hydrogel-coated wells. The lower compartment contained medium supplemented with 10% FBS (Welgene) and 1% penicillin–streptomycin (Thermo Fisher Scientific) as a chemoattractant. Cells were incubated at 37 °C in a humidified atmosphere with 5% CO_2_. After 18 h, the hydrogels were carefully removed by sectioning, and cells that had invaded through and adhered to the bottom of the wells were fixed with 100% methanol at −20 °C. Fixed cells were stained with 0.5% crystal violet solution (Sigma-Aldrich, St. Louis, MO, USA) for 10 min and gently washed with PBS. The number of invading cells was quantified by counting five random microscopic fields per well using a phase-contrast microscope (EVOS M7000, Thermo Fisher Scientific). Each experiment was performed in triplicate to ensure reproducibility.

### 4.8. RNA Isolation and cDNA Synthesis

Total RNA was extracted from HLF, Huh7, and HepG2 cells cultured under 2D or 3D conditions for 5 or 7 days using TRIzol reagent (Favorgen Biotech Corp., Pingtung, Taiwan), following the manufacturer’s instructions. RNA quality and concentration were assessed with a NanoDrop 2000 spectrophotometer (Thermo Fisher Scientific), and only samples with acceptable purity ratios (A260/A280) were used for downstream analysis. For cDNA synthesis, 1 µg of total RNA per sample was reverse-transcribed using the HiSenScript™ RH (−) RTase cDNA Synthesis Kit (iNtRON Biotechnology, Seongnam, Republic of Korea). Reaction was carried out at 45 °C for 60 min, followed by heat inactivation at 85 °C for 10 min.

### 4.9. Quantitative Real-Time PCR (qRT-PCR)

Quantitative real-time PCR was performed using the CFX Connect Real-Time PCR Detection System (Bio-Rad, Hercules, CA, USA) with SsoAdvanced Universal SYBR Green Supermix (Bio-Rad). Primer sequences are provided in Table 1. Each reaction was performed in triplicate to ensure reproducibility. Gene expression was quantified using the 2^−ΔΔCt^ method, with GAPDH as the internal control. Expression levels were normalized to the control sample (set to 1), and relative fold changes in experimental groups were calculated.

### 4.10. Flow Cytometry

To identify CSC populations, HLF, HepG2, Huh7, and PLC/PRF/5 cells were cultured under 2D and 3D conditions for 7 days and harvested by gentle pipetting. Cells were washed with Hanks’ balanced salt solution (HBSS; Thermo Fisher Scientific) containing 0.1% bovine serum albumin (BSA; Sigma-Aldrich) and 0.1% sodium azide (Sigma-Aldrich), then filtered through a 100-μm cell strainer (SPL Life Sciences) to remove aggregates.

For phenotypic analysis, cells were washed twice with HBSS and resuspended in cell-staining buffer. Anti-ALDHA1 antibody (1:100; BD Biosciences, San Jose, CA, USA) was incubated overnight at 4 °C. Anti-CD44 (1:100; BioLegend, San Diego, CA, USA), anti-Cytokeratin 19 (1:100; BioLegend), and anti-EpCAM (1:100; BioLegend) were incubated for 1 h at room temperature. After washing, cells were incubated for 1 h with anti-rat and anti-mouse secondary antibodies (BioActs, Incheon, Republic of Korea).

In a separate experiment, cells were directly stained with FITC-labeled anti-CD90 (1:10; BioLegend) and PE-labeled anti-CD117 (1:10; BioLegend) monoclonal antibodies for 25 min at room temperature. Flow cytometric acquisition was performed using a FACS Symphony A3 cytometer (BD Biosciences), and data were analyzed with FlowJo software, version 10.10.0 (Tree Star, Ashland, OR, USA).

### 4.11. Cell Cycle Analysis

For flow cytometric analysis of the cell cycle, HLF and HepG2 cells were cultured under 2D and 3D conditions for 7 days and harvested by gentle pipetting. Cells were washed with HBSS containing 0.1% BSA and 0.1% sodium azide, then fixed with 70% ethanol at −20 °C for at least 2 h or overnight. Following fixation, cells were washed with HBSS and resuspended in staining buffer. They were incubated with 50 μg/mL PI (BioLegend) for 30 min in the dark to stain DNA. Samples were acquired on a FACS Symphony A3 flow cytometer (BD Biosciences), and cell cycle distribution (G0/G1, S, and G2/M phases) was analyzed with FlowJo software, version 10.10.0 (Tree Star, Ashland, OR, USA).

### 4.12. Cell Spheroid-Based Anti-Cancer Drug Test

To assess the antitumor efficacy of various chemotherapeutic agents in a 3D spheroid culture system using MCP-B hydrogel, cancer cell spheroids were seeded in 96-well plates (1 × 10^4^ cells/well) and allowed to stabilize for 24 h. Following spheroid establishment, cells were treated with different concentrations of each drug in serum-free medium for 24 h. The following agents were tested: sorafenib (MedChemExpress, Monmouth Junction, NJ, USA; 0, 1, 5, 10, and 25 μM); cisplatin (Sigma-Aldrich; 0, 10, 25, 50, and 100 μM); curcumin (Sigma-Aldrich; 0, 25, 50, 75, and 100 μM); paclitaxel (Enzo Life Sciences, Farmingdale, NY, USA; 0, 10, 50, 100, and 150 nM); docetaxel (Sigma-Aldrich; 0, 1, 10, 50, and 100 nM); doxorubicin (Cell Signaling Technology, Danvers, MA, USA; 0, 1, 10, 25, and 50 μM); 5-fluorouracil (Sigma-Aldrich; 0, 10, 50, 100, and 200 μM); cabazitaxel (Sigma-Aldrich; 0, 1, 10, 50, and 100 nM); cabozantinib (Selleckchem, Houston, TX, USA; 0, 1, 10, 50, and 100 μM); and mitoxantrone (Selleckchem; 0, 1, 5, 10, and 20 μM).

Spheroid size was evaluated using a phase-contrast microscope (EVOS M7000, Thermo Fisher Scientific). For each condition, at least five spheroids per hydrogel were imaged, and their diameters were measured. Cell viability was determined 24 h after drug treatment using the WST-1 colorimetric assay, following the manufacturer’s protocol. Formazan absorbance was measured at 450 nm with a microplate reader (Tecan, Männedorf, Switzerland), and viability was expressed as a percentage relative to untreated controls.

To further evaluate drug response, dose–response curves were generated for each compound, and half-maximal inhibitory concentration (IC_50_) values were calculated in Excel as the concentration producing 50% inhibition of viability. This analysis enabled quantitative comparison of drug sensitivity across different spheroid cultures. All experiments were performed independently in triplicate to ensure reproducibility.

### 4.13. Western Blot Analysis

Protein expression was assessed by Western blotting. Cells were harvested and washed twice with ice-cold PBS, then lysed in radioimmunoprecipitation assay buffer (GenDEPOT, Barker, TX, USA) supplemented with a protease inhibitor cocktail (GenDEPOT). Lysis was carried out on ice for 40 min with vortexing every 10 min. Lysates were centrifuged at 13,000 rpm for 30 min at 4 °C, and supernatants were collected. Protein concentrations were determined using the bicinchoninic acid (BCA) protein assay (Sigma-Aldrich).

Equal amounts of protein (12 μg per sample) were mixed with Laemmli sample buffer (Bio-Rad, Hercules, CA, USA), resolved by 10% SDS–PAGE, and transferred to polyvinylidene fluoride (PVDF) membranes (Amersham Biosciences, Piscataway, NJ, USA) using a semi-dry transfer system (Bio-Rad). Membranes were blocked with 3% BSA in Tris-buffered saline containing 0.1% Tween-20 (TBST) for 1 h at room temperature.

Membranes were incubated overnight at 4 °C with primary antibodies against Bax (Ab262929, Abcam, Cambridge, UK), Bad (Ab90435, Abcam), Bcl-xL (2764S, Cell Signaling Technology), Cleaved PARP (9541S, Cell Signaling Technology), fibronectin (Ab2413, Abcam), and β-actin (Sc-47778, Santa Cruz Biotechnology, Dallas, TX, USA). After washing, membranes were incubated for 1 h at room temperature with HRP-conjugated secondary antibodies (anti-rabbit 7074S; anti-mouse 7076P2; Cell Signaling Technology).

Protein bands were visualized using an enhanced chemiluminescence (ECL) kit (Dongin Biotech, Seoul, Republic of Korea), imaged using the Amersham Imager 680 (Amersham Biosciences), and quantified with ImageJ software (version 1.52a, National Institutes of Health, Bethesda, MD, USA).

### 4.14. Statistical Analysis

All quantitative data are presented as the mean ± SD from at least three independent experiments. Statistical comparisons between two groups were performed using the unpaired Student’s *t*-test. A *p*-value < 0.05 was considered statistically significant.

## 5. Conclusions

We established an effective in vitro 3D HCC model using the MCP-B hydrogel matrix, specifically developed and optimized to support multicellular HCC spheroid growth across diverse cell lines. This system offers several advantages over conventional approaches, including simplicity, reproducibility, bioactivity, efficiency, and low cost, making it suitable for both basic research and translational applications. Notably, HCC cells cultured within the MCP-B hydrogel recapitulated key biochemical and physiological hallmarks of tumor biology. The 3D culture system promoted malignant behaviors such as increased proliferation, migration, invasion, and colony formation, while conferring resistance to chemotherapy through upregulation of multidrug resistance genes (MDR1, MRP1, ABCG2). Liver-specific functionality was retained, as shown by elevated albumin, CYP1A1, CYP1A2, CYP3A4, and HNF4A expression. The system also enriched cancer stem-like properties, with higher expression of stemness-associated genes (CD24, CD44, CD133, ALDHA1, CXCR4, Sox2, Oct4, Nanog, and KLF4) and expansion of CSC populations expressing (CD44, CD90, CD117, EpCAM, and CK19). EMT programs were strongly activated, with upregulation of EMT markers (vimentin and N-cadherin), transcription factors (Snail, Slug, Twist, Zeb1, and Zeb2), and matrix-remodeling enzymes (MMP2 and MMP9). Oncogenic signaling was also reinforced, including Notch-1/2 and growth factor pathways (HGF, IGF1, IGF2, VEGFA, and TGFβ) with their receptors (c-Met, IGF1R, IGF2R, and TGFβR). Finally, ECM and fibrosis-related molecules (COL1A1, COL4A1, COL6A3, fibronectin, and LAMC1) were elevated, closely mimicking the desmoplastic tumor microenvironment of HCC. A limitation of this study is that mRNA expression changes were not validated at the protein or functional level. Future studies should validate these findings at the protein and functional levels to better define the physiological relevance of MCP-B hydrogels in 3D HCC culture. Collectively, these results demonstrate that the MCP-B hydrogel-based 3D HCC culture system is a robust and physiologically relevant in vitro platform, well suited for investigating HCC progression, liver CSC biology, and screening novel anti-HCC and anti-CSC therapeutics.

## Figures and Tables

**Table 1:** qRT-PCR primer names and their sequences.

Gene Name	Forward (5′–3′)	Reverse (5′–3′)
*ABCG2*	GTTCTCAGCAGCTCTTCGGCTT	TCCTCCAGACACACCACGGATA
*Albumin*	TTTATGCCCCGGAAGTCCTTT	AGTCTCTGTTTGGCAGACGAA
*ALDHA1*	CGGGAAAAGCAATCTGAAGAGGG	GATGCGGCTATACAACACTGGC
*CD24*	CACGCAGATTTATTCCAGTGAAAC	GACCACGAAGAGACTGGCTGTT
*CD44*	CTGCCGCTTTGCAGGTGTA	CATTGTGGGCAAGGTGCTATT
*CD133*	CACTACCAAGGACAAGGCGTTC	CAACGCCTCTTTGGTCTCCTTG
*c-Met*	AGGCTTCTGGGCCTTAT	TGCTTCTCTCGCCAGGAATAC
*COL1A1*	ATTAGTAGGTGTGCTGTGTG	AAGCGTTTGCGTAGTAATTG
*COL4A1*	ATTAGTAGGTGTGCTGTGTG	AAGCGTTTGCGTAGTAATTG
*CYP1A1*	TGGATGAGAACGCCAATG	TGGGTTGACCCATAGCTTCT
*CYP1A2*	AACAAGGGACACAACGCTGAAT	GGAAGAGAAACAAGGGCTGAGT
*CYP3A4*	TTTTGTCCTACCATAAGGGC	CATAAATCCCACTGGACCAA
*CXCR4*	CTCCTCTTTGTCATCACGCTTCC	GGATGAGGACACTGCTGTAGAG
*Fibronectin*	ACCTACAACATCATAGTGGAGGCACTG	GTCACAGCGCCAGCCCCGCTGGCCTCC
*HGF*	CTCACACCCGCTGGGAGTAC	TCCTTGACCTTGGATGCATTC
*HNF4A*	GGCCAAGTACATCCCAGCTTT	CAGCACCAGCTCGTCAAGG
*IGF1*	GCTCTTCAGTTCGTGTGTGG	CGCAATACATCTCCAGCCTC
*IGF2*	GGGCAAGTTCTTCCAATATGA	TCACTTCCGATTGCTGGC
*IGF1R*	AACCCCAAGACTGAGGTGTG	TGACATCTCTCCGCTTCCTT
*IGF2R*	GGCACAATTACTGCTCCAAAGAC	CAAGGCCCTTTCTCCCCAC
*KLF4*	CATCTCAAGGCACACCTGCGAA	TCGGTCGCATTTTTGGCACTGG
*LAMC1*	ACTCCTAATCTTGGACCATAC	ACAACAGCACAACTTGAAC
*MDR1*	GCTGTCAAGGAAGCCAATGCCT	TGCAATGGCGATCCTCTGCTTC
*MMP2*	TGACGGTAAGGACGGACTC	ATACTTCACACGGACCACTTG
*MMP9*	CAGAGATGCGTGGAGAGT	TCTTCCGAGTAGTTTTGG
*MRP1*	CCGTGTACTCCAACGCTGACAT	ATGCTGTGCGTGACCAAGATCC
*Nanog*	CTCCAACATCCTGAACCTCAGC	CGTCACACCATTGCTATTCTTCG
*N-cadherin*	AGCCAACCTTAACTGAGGAGT	GGCAAGTTGATTGGAGGGATG
*Notch1*	GGTGAACTGCTCTGAGGAGATC	GGATTGCAGTCGTCCACGTTGA
*Notch2*	GTGCCTATGTCCATCTGGATGG	AGACACCTGAGTGCTGGCACAA
*Oct4*	CTTGAATCCCGAATGGAAAGGG	GTGTATATCCCAGGGTGATCCTC
*Snail*	ACTGCAACAAGGAATACCTCAG	GCACTGGTACTTCTTGACATCTG
*Slug*	TGTGACAAGGAATATGTGAGCC	TGAGCCCTCAGATTTGACCTG
*Sox2*	GCTACAGCATGATGCAGGACCA	TCTGCGAGCTGGTCATGGAGTT
*Twist*	GTCCGCAGTCTTACGAGGAG	GCTTGAGGGTCTGAATCTTGCT
*VEGFA*	TTGCCTTGCTGCTCTACCTCCA	GATGGCAGTAGCTGCGCTGATA
*Vimentin*	CAAAGCAGGAGTCCACTGAG	TAAGGGCATCCACTTCACAG
*TGFβ*	GGGACTATCCACCTGCAAGA	CCTCCTTGGCGTAGTAGTCG
*TGFβR*	GACAACGTCAGGTTCTGGCTCA	CCGCCACTTTCCTCTCCAAACAACT
*Zeb1*	TTACACCTTTGCATACAGAACCC	TTTACGATTACACCCAGACTGC
*Zeb2*	GCGATGGTCATGCAGTCAG	CAGGTGGCAGGTCATTTTCTT
*GAPDH*	GGAGAAGGCTGGGGCTCAT	TGATGGCATGGACTGTGGTC

## Data Availability

The data presented in this study are available on request from the corresponding author.

## Abbreviations

HCC	Hepatocellular carcinoma
MCP-B	Marine collagen peptides-based
ECM	Extracellular matrix
EMT	Epithelial–mesenchymal transition
CSC	Cancer stem cell
MASLD	Metabolic dysfunction-associated steatotic liver disease
MDR1	Multidrug resistance protein 1
MRP1	Multidrug resistance-associated protein 1
CYP	Cytochrome P450
